# Identification of cecum time-location in a colonoscopy video by deep learning analysis of colonoscope movement

**DOI:** 10.7717/peerj.7256

**Published:** 2019-07-29

**Authors:** Minwoo Cho, Jee Hyun Kim, Kyoung Sup Hong, Joo Sung Kim, Hyoun-Joong Kong, Sungwan Kim

**Affiliations:** 1 Interdisciplinary Program for Bioengineering, Graduate School, Seoul National University, Seoul, South Korea; 2 Department of Gastroenterology, Seoul National University Boramae Medical Center, Seoul, South Korea; 3 Department of Gastroenterology, Mediplex Sejong Hospital, Incheon, South Korea; 4 Department of Internal Medicine, Seoul National University College of Medicine, Seoul, South Korea; 5 Department of Biomedical Engineering, Chungnam National University College of Medicine, Daejeon, South Korea; 6 Department of Biomedical Engineering, Seoul National University College of Medicine, Seoul, South Korea

**Keywords:** Colonoscopy, Cecum, Cecal-location, Summary report, CNN, Deep learning

## Abstract

**Background:**

Cecal intubation time is an important component for quality colonoscopy. Cecum is the turning point that determines the insertion and withdrawal phase of the colonoscope. For this reason, obtaining information related with location of the cecum in the endoscopic procedure is very useful. Also, it is necessary to detect the direction of colonoscope’s movement and time-location of the cecum.

**Methods:**

In order to analysis the direction of scope’s movement, the Horn–Schunck algorithm was used to compute the pixel’s motion change between consecutive frames. Horn–Schunk-algorithm applied images were trained and tested through convolutional neural network deep learning methods, and classified to the insertion, withdrawal and stop movements. Based on the scope’s movement, the graph was drawn with a value of +1 for insertion, −1 for withdrawal, and 0 for stop. We regarded the turning point as a cecum candidate point when the total graph area sum in a certain section recorded the lowest.

**Results:**

A total of 328,927 frame images were obtained from 112 patients. The overall accuracy, drawn from 5-fold cross-validation, was 95.6%. When the value of “t” was 30 s, accuracy of cecum discovery was 96.7%. In order to increase visibility, the movement of the scope was added to summary report of colonoscopy video. Insertion, withdrawal, and stop movements were mapped to each color and expressed with various scale. As the scale increased, the distinction between the insertion phase and the withdrawal phase became clearer.

**Conclusion:**

Information obtained in this study can be utilized as metadata for proficiency assessment. Since insertion and withdrawal are technically different movements, data of scope’s movement and phase can be quantified and utilized to express pattern unique to the colonoscopist and to assess proficiency. Also, we hope that the findings of this study can contribute to the informatics field of medical records so that medical charts can be transmitted graphically and effectively in the field of colonoscopy.

## Introduction

A polyp is an abnormal tissue growth and is commonly found in the intestine (Haggar & Boushey, 2009). Since all colon and rectal cancers arise from a polyp, it is crucial to detect polyps in the early stage and treat them before they progress to being cancerous (Leslie et al., 2002). Colonoscopy is the most commonly used method to detect polyps and most available method (Nishihara et al., 2013; Rex, 2002). For this reason, the demand for colonoscopy continues to increase (Quintero et al., 2012).

Cecal intubation time (CIT) provides various data, which may be important indicators (Marshall & Barthel, 1993; Bernstein et al., 2005). In addition, when performing colonoscopy, the cecum is the turning point that determines the insertion phase and withdrawal phase of the colonoscope, that is, the gastroenterologist inserts the colonoscope close to the appendix and withdraws it from the cecum (Fatima et al., 2008). However, there are individual (i.e., patient and gastroenterologist) differences in the sequence and process of advancing the colonoscopy (Spier et al., 2010; Rex, 2001; Saifuddin et al., 2000). Thus, obtaining information about the time-location of the cecum in the colonoscopic procedure is very useful.

Information about the time-location of the cecum can also be helpful when checking colonoscopy videos. Because analyzing a video requires much time and concentration, it places a great burden on the physician, especially when the doctor re-watches the video or shares the video due to change of doctor or hospital (Terada, 2015; Hu et al., 2016). Knowing the time-location of the cecum can help physicians to distinguish the insertion phase and the withdrawal phase of the colonoscope (Fatima et al., 2008), which can help reduce the burden on video observation. In addition, because detailed examination was undertaken mostly during the withdrawal phase of the scope after reaching the cecum, knowing the time-location of the cecum and distinction between the insertion phase and withdrawal phase of the colonoscope is important (Barclay et al., 2006; Barclay, Vicari & Greenlaw, 2008; Moritz et al., 2012). Furthermore, the CIT and withdrawal phase can also be useful as metadata (Taber & Romagnuolo, 2010; Lee et al., 2009). The location of other anatomic sites such as the T-colon and S-colon can be inferred on the assumption that the location of the cecum is known (Cherian & Singh, 2004).

Moreover, the movement data of the colonoscope can be utilized for proficiency assessment (Lee et al., 2009; Snyder et al., 2010). Since insertion and withdrawal are technically different movements, the pattern, combination, and repetition of insertion/withdrawal/stop can be utilized to express individual features of the colonoscopist and to assess proficiency (Marshall, 1995; Benson et al., 2010; Anderson et al., 2001). Thus, it is necessary to detect the direction of the scope’s movement and time-location information of the cecum.

Recently, with advances in computer technology and equipment, gastroenterologists do not need to record these data anymore (Denny et al., 2010; Leiman et al., 2016). In various medical fields, systems that automatically record medical reports are being developed (Münzer, Schoeffmann & Böszörmenyi, 2018; Yuan, Li & Meng, 2016; Greenhalgh et al., 2010; Taira, Soderland & Jakobovits, 2001). In our previous study, we developed a useful system that automatically extracts meaningful information (namely, bleeding, polypectomy, tool, residue, thin wrinkle, folded wrinkle) from colonoscopy videos using support vector machine (SVM) and provides such information on the summary report with color-coded timeline visualization (Cho et al., 2018).

Horn–Schunk algorithms are the most popular differential algorithms that have been used for many applications and have been referenced for many performance evaluation models (Meinhardt-Llopis, Pérez & Kondermann, 2013). The Horn–Schunk algorithm is a technique used to identify the image velocity or motion vector based on spatial temporal gradient technique that computes the image velocity from spatiotemporal derivatives of the image intensity (Gong & Bansmer, 2015; Horn & Schunck, 1981). Through this algorithm, we can compute the pixel’s motion change between consecutive frames (Bruhn, Weickert & Schnörr, 2005). In other words, it is possible to know the movement direction of the pixels in the current frame relative to the previous frame; thus, it is possible to determine the direction in which the current frame is moving.

Therefore, in this study, we aimed to provide more useful information to physicians by recording the direction of the scope’s movement and time-location of the cecum using Horn–Schunk algorithm by applying convolutional neural network (CNN).

## Material & Methods

This study was approved by the institutional review board of the Seoul National University Hospital (IRB No. 1509-062-703), and it was conducted in accordance with the Declaration of Helsinki. Informed consent was obtained from all participants before any study-related procedures were performed. This prospective, single-center trial enrolled patients aged 19 to 75 years who underwent colonoscopy for screening, surveillance, or therapy such as polypectomy at Seoul National University Hospital, a tertiary referral center in Korea, from August 2016 to December 2016.

### Acquisition of colonoscopy video

In this study, colonoscopy was performed using a high-resolution colonoscopy device (CV260SL, Olympus, Tokyo, Japan). Colonoscopy videos were acquired using a video capture card (SkyCaputre U6T; Skydigital, Seoul, Korea), after signal branching from the CV260SL.

The video was converted to an MP4 format to avoid alteration of the resolution, and the resolution was 1920*1080, 30fps. Videos were acquired from 112 patients, and the play time was about 20-40 min. The colonoscopy video was decomposed into frames. A frame was extracted as a PNG file per 0.5 s using Virtualdub software. All frames were cropped to 850*750 pixels to extract only the colonoscopy area, excluding patient information and the settings.

### Classification of informative frames

As the play time of the colonoscopy videos was long and had much noise, it was inefficient to use the whole video (Vilariño et al., 2007). Thus, frames were extracted to select only meaningful information or applied to image processing. In the colonoscopy video, the frame that has meaningful information is called informative frame, and the other is called non-informative frame (Vilariño et al., 2007). Since non-informative frames are frames that do not have meaningful information in the image itself, they have had a negative effect on training (Ballesteros et al., 2016). In this study, only informative frames were used. The classification accuracy of the informative frame used in this study was 99.4%.

#### Horn–Schunk algorithm

As the Horn–Schunk algorithm can only estimate small motions and compares the current frame with the previous frame to calculate the motion vector, the time interval between the two frames has a significant effect on the outcome (Meinhardt-Llopis, Pérez & Kondermann, 2013; Horn & Schunck, 1981). If the interval is too large, the calculation error will increase beyond the small motion, and if the interval is too small, the movement of the scope will not be considered sufficiently. Considering this limitation, we extracted the frame per 0.5 s.

**Figure 1:**
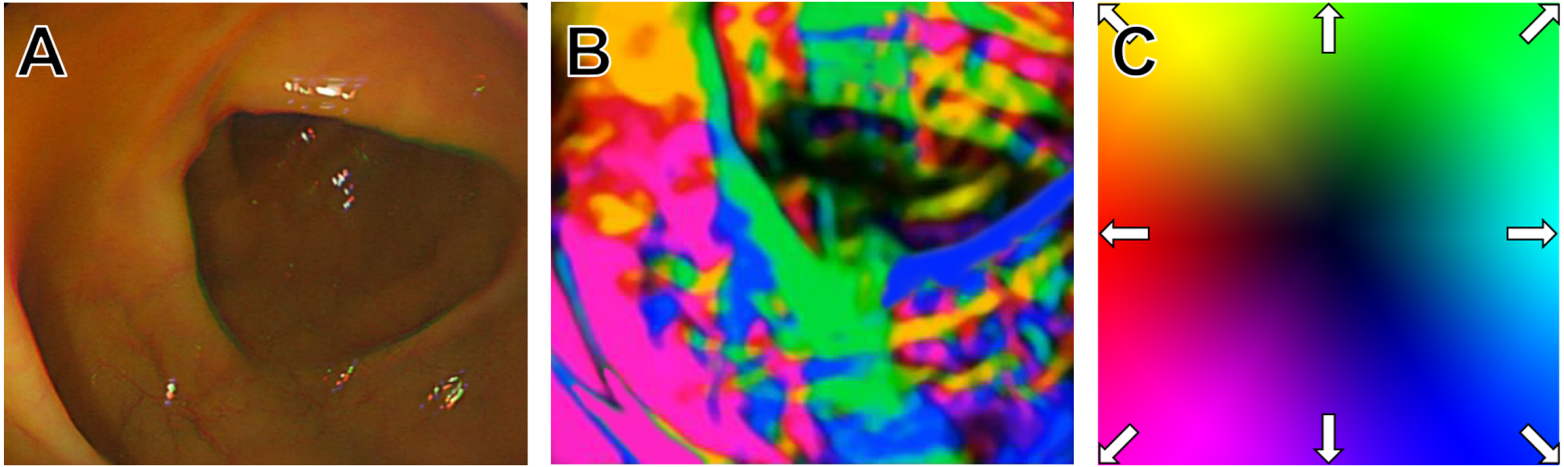
Horn and Schunk algorithm application. (A) Original image frame, (B) Horn and Schunk algorithm applied image, (C) motion vector and its color expression.

Because the image inside the large intestine had varied shapes and colors depending on the conditions of position, health status, and patient’s condition, it is advantageous to remove other information from the frame image for training, leaving only information about the position using Horn-Schunck algorithm. After Horn-Schunck algorithm application, we acquired the image shown in Fig. 1 by expressing the direction change of the pixels inside the frame in color. Since all elements inside the frame do not move in the same direction consistently, various colors appear mixed, as shown in Fig. 1. To understand the pattern of colors according to these positions, we used CNN deep learning in the next step.

### Three-direction classification of informative frame

**Figure 2:**
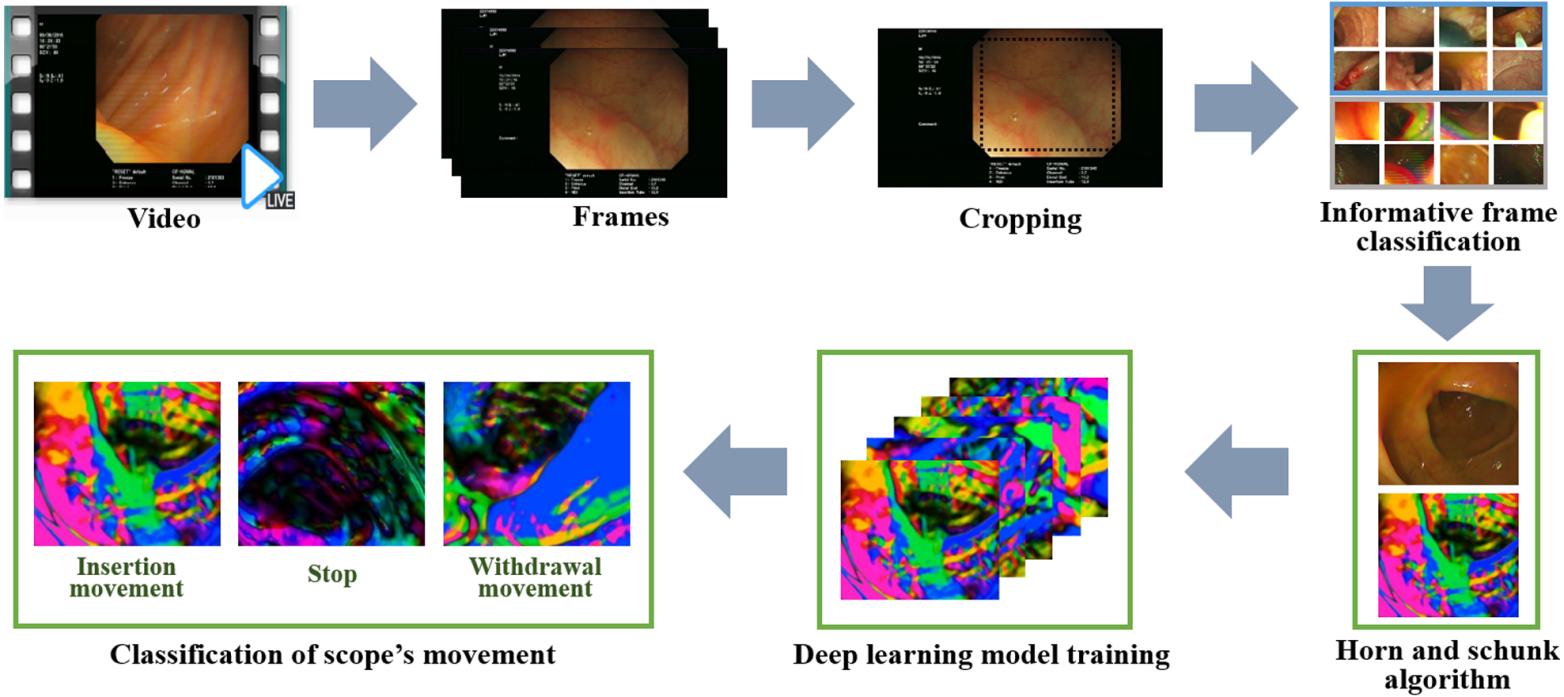
Overview and process of the proposed system. After extracting the frame from the video, only the informative frame is extracted by the SVM. In the informative frame, the motion vector is expressed in color by Horn and Schunk algorihm. Through the CNN deep learning method, these color images were classified into three types: insertion, withdrawal, and stop.

In this paper, Horn-Schunck algorithm-applied color images were classified into three types: insertion, withdrawal, and stop. Therefore, frames from colonoscopy images of 112 patients were classified as insert, withdrawal, and stop as the standard for deep training set. To make a standard setting, a colonoscopy video was reproduced at a speed of 0.7 times, and a user observing it pressed the direction key of the keyboard in real time. The up, down, and space buttons were matched to insert, withdrawal, and stop, respectively, and the frames were classified based on the input keyboard values. Five gastroenterologist with more than 5 years experience participated in this work, and the values selected by more than three were used as the standard (Fallah & Niakan Kalhori, 2017). An overview of the entire process is shown in Fig. 2.

### CNN machine learning for three-direction classification

CNN deep learning was applied based on the standard created above (Le, Ho & Ou, 2017; Ho & Ou, 2018; Taju et al., 2018). Horn-Schunck algorithm-applied color images were used as training data and test data. Unlike previous research using SVM, we used CNN, a deep learning technique, because it was difficult to select features from the Horn-Schunck algorithm-applied color image (Rajaraman et al., 2018).

**Figure 3:**
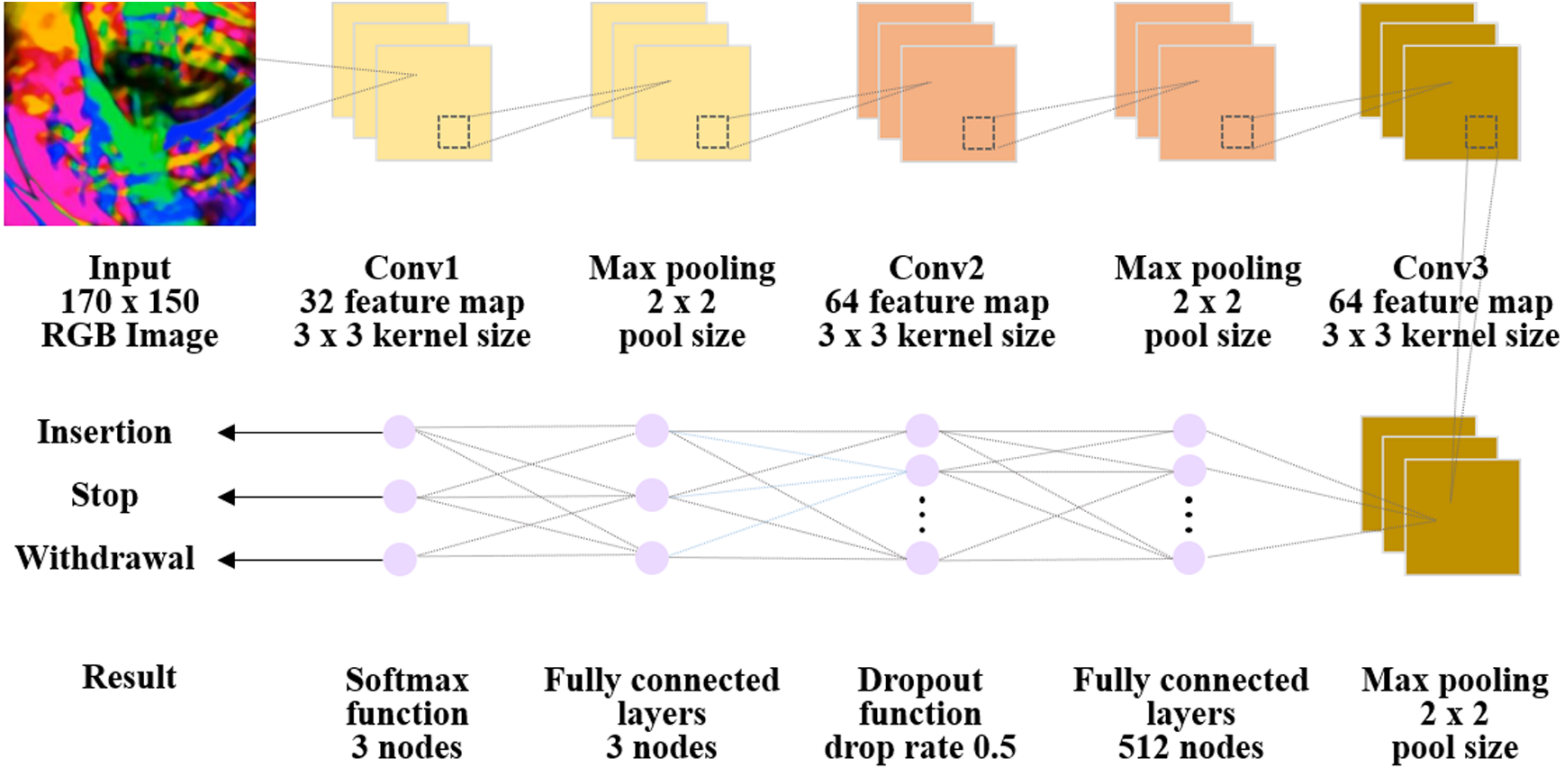
Overall architecture of the convolutional neural network (CNN) deep learning model. All image were reduced to 170 × 150 and were input into the model. Next, it was passed through all convolution layers and the entire binding layer, and it was classified into three classes.

The CNN structure and training procedure are described in Fig. 3. The network architecture consisted of an input stage, a feature extraction stage with three convolutional layers, and an output classification stage (Nagasawa et al., 2018; Jang et al., 2018). The input stage received the Horn-Schunck algorithm applied color image which was converted to 170*150 pixels. The feature extraction stage that followed a triple ternary convolution block structure was composed of convolution, pooling, and activation functions. The rectified linear unit (ReLU) activation function and max polling layer were placed after each convolutional layer. The classification stage included fully connected layers and a dropout function (drop rate of 0.5) and provided an output from a Softmax function. Fully connected layer were classified into three classes using the Softmax function.

The learning was carried out with batch size 128 (set by experimental trials) images. The model is optimized for hyper-parameters by a randomized grid search method (Bergstra & Bengio, 2012). We initialized search ranges to be [1e–7 1e–2], [0.8 0.99], and [1e–10 1e–2] for the learning rate, SGD, and L2-regularization parameters, respectively.

Finally, the trained network was validated using the 5-fold cross-validation method (Ou, 2016; Ou, 2017; Le, Sandag & Ou, 2018). Five groups of data were established such that four groups were used for training and the remaining groups were used for validation. Each group was designed to participate in validation in turn. The performance was measured using the mean of the validation results for each group.

The model was trained and tested on a Windows 10 Pro system with Intel i9-9900K CPU 3.6-GHz processor, 2TB SDD, 64 GB RAM, GeForce RTX 2070 Gaming OC D6 8GB.

### Cecum time-location calculation

To determine the location of the cecum, we analyzed the direction of the scope’s movement. Based on the results of the analysis, the graph was drawn with a value of +1 for insertion, −1 for withdrawal, and 0 for stop. Because the cecum is a large turning point that distinguishes the insertion phase from the withdrawal phase, the turning point of transition from insertion to withdrawal is the candidate for the cecum. However, some scopic insertion-withdrawal repeat movements often occur during colonoscopy, and turning points can be found very often. Therefore, in this study, the scope movement for a certain range was analyzed to find the real turning point. All points that transitioned from insertion to withdrawal were recorded. These turning points were referred to as a cecum candidate point.

**Figure 4:**
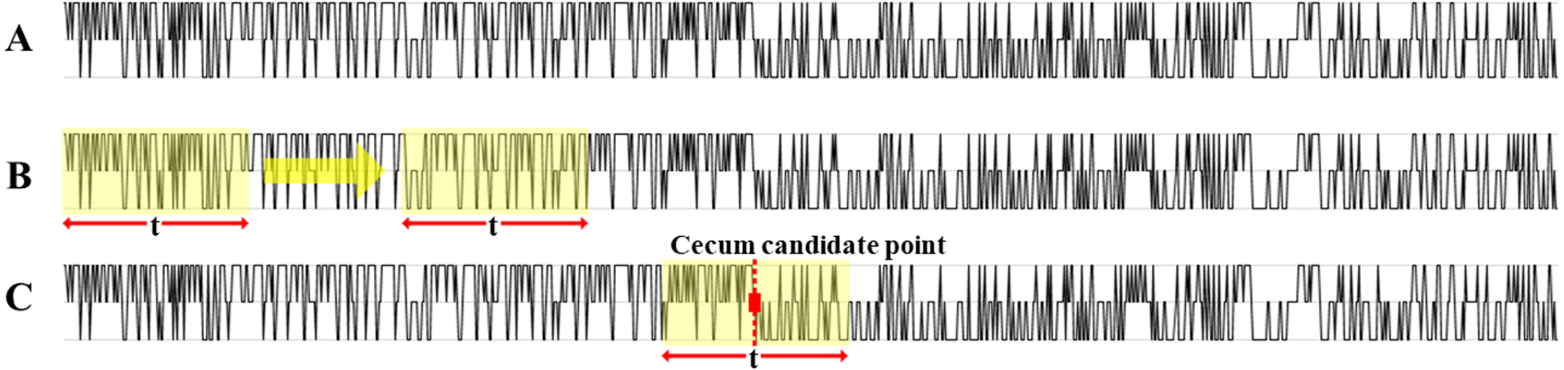
The movement of the scope. (A) Original curve graph of insertion and withdrawal movement, (B) graph area calculation for cecal position prediction, (C) cecum candidate point.

We set a certain range “t” and calculated the sum of the graph area $\pm \frac{t}{2} $ around the turning points. Since the value is +1 during insertion, the area of the graph will be positive, and when the value is −1 during withdrawal, the area of the graph will be negative. At the turning point with a certain range “t,” when the total sum of the graph areas is the minimum value, that turning point is regarded as the cecum (Fig. 4). If the value “t” is too small, light repetitive movements of insertion-withdrawal may have a minimum area value, so “t” is set to a sufficiently large value. For several candidate points, the graph area was calculated based on range “t.” The range “t” was set to 10, 20, and 30 s.

### Focus group interview (FGI) for the proposed system

In this study, we proposed the system that can analyze the colonoscopy video and provide meaningful direction information of endoscope’s movement and time-location information of the cecum via visualized summary report. To ask for comments on the system and reflect gastroenterologists’ requirements, we had implemented a FGI. Seven gastroenterologists with more than 5 years experience participated in this FGI, and it was conducted in the face-to-face meeting. The comments on necessity referred in this research were selected by FGI, and ideas about visualization were also recruited and validated. This FGI simply asked positive and negative opinions, and the results are as follows. All seven doctors determined that this system would positively benefit physicians and patients. Six doctors agreed that a visualized summary report based on this system would be helpful for medical records management and data sharing. Five doctors agreed that the results could be used for proficiency testing.

## Results

In this study, we trained and tested the CNN models using 328,927 frames from 112 colonoscopy videos. To facilitate physician’s diagnosis during colonoscopy, we recruited most patients receiving colonoscopy for the second time.

### Classification according to direction of scope’s movement

Since the features of Horn–Schunk-algorithm applied images were difficult to specify, we applied the CNN deep learning method to the images for high accuracy. The overall accuracy, drawn from 5-fold cross-validation, was 95.6%. Table 1 shows the classification results in confusion matrix form. Each cell in the Table 1 represents the number of samples classified by the trained CNN algorithm. The vertical title indicates the actual class (input class of the algorithm), whereas the horizontal title indicates the classified results (output class of the algorithm). Performance comparisons according to the indicators of recall, precision, and f1 score for each class are presented in Table 2. These numbers can be used for alternative algorithm assessments (Kainz, Pfeiffer & Urschler, 2017).

**Table 1:** The confusion matrix of classification results using the 5-fold cross-validation of the CNN algorithm.

		Target class			Sum by row
		Insertion	Withdrawal	Stop	
Output class	Insertion	116,437	1,533	1,379	119,349
	Withdrawal	1,586	107,336	1,757	110,679
	Stop	3,484	4,603	90,812	98,899
Sum by column		121,507	113,472	93,948	328,927

**Table 2:** Performance indicators of the CNN and other algorithms for the three individual classes.

**Class/Indicators**		Insertion	Withdrawal	Stop
CNN	Recall	0.958	0.946	0.967
	Precision	0.976	0.969	0.918
	F1 score	0.967	0.958	0.942
VGG-16	Recall	0.95	0.916	0.964
	Precision	0.953	0.959	0.911
	F1 score	0.952	0.937	0.937
LeNET	Recall	0.907	0.833	0.872
	Precision	0.874	0.909	0.83
	F1 score	0.89	0.87	0.85
SVM	Recall	0.844	0.775	0.799
	Precision	0.804	0.832	0.785
	F1 score	0.823	0.802	0.792

### Cecum time-location calculation

**Figure 5:**
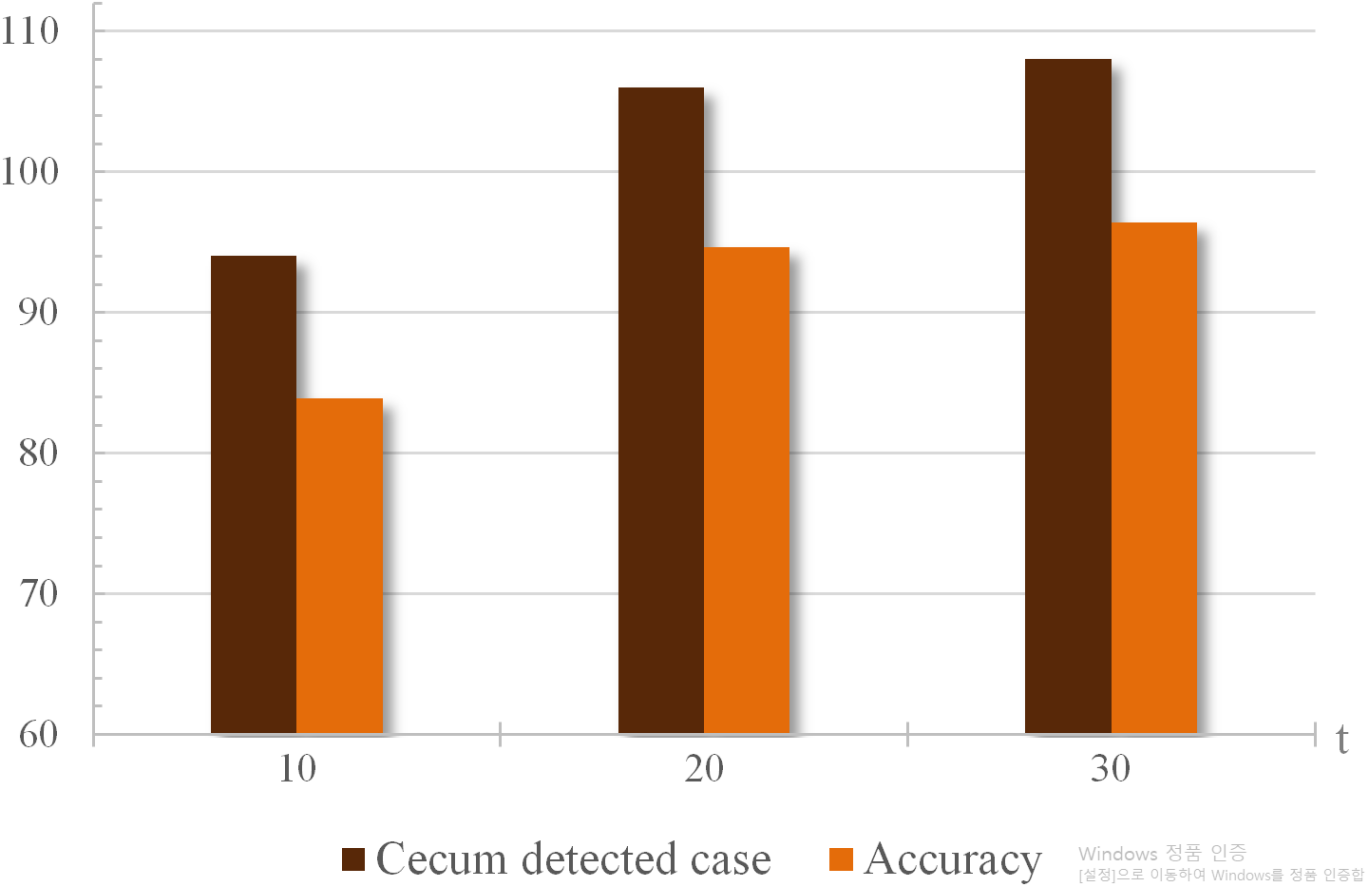
Accuracy and number of cases when cecum is detected according to “t” values ranging from 10 s to 30 s. If “t” is less than 10 s, light repetitive insertion-withdrawal movements may have a minimum area value, so “t” is set to a sufficiently large value. Considering this, we analyzed the results when t values were 10, 20, and 30 s.

We regarded the turning point as a cecum candidate point when the total area sum in a certain section recorded the lowest. We checked the existence of the actual cecum within a certain interval $\pm \frac{t}{2} $ around the cecum candidate point. The accuracy according to “t” values is shown in Fig. 5 and Table 3. The position of the cecum recorded by three clinicians was compared with the results of the proposed system.

**Table 3:** Accuracy of cecum discovery according to the set certain range “t”.

	t = 10	t = 20	t = 30
Accuracy	**83.9%**	**94.6%**	**96.4%**

### Summary reports visualized in the timeline

**Figure 6:**
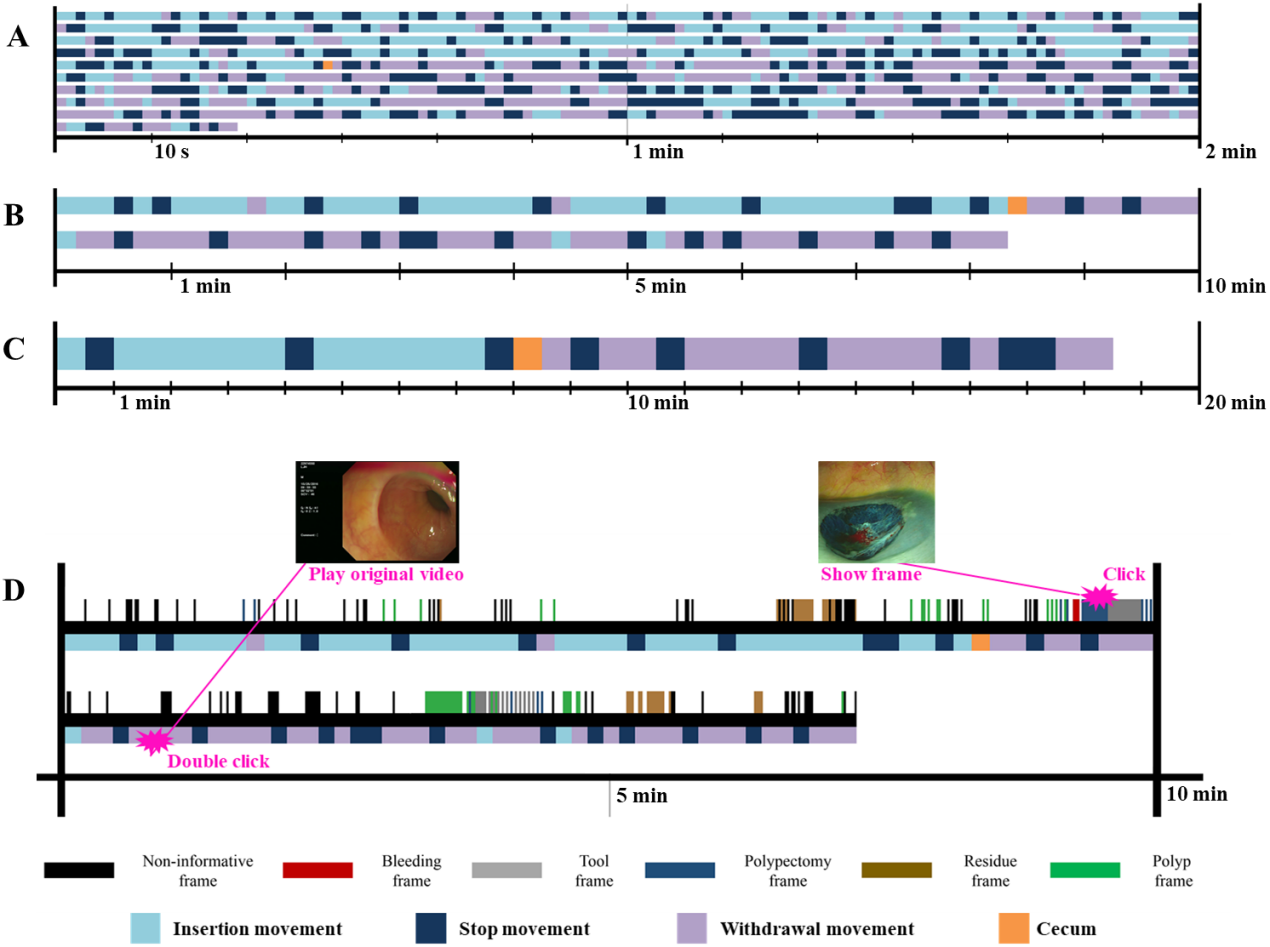
Insertion, withdrawal, and stop were mapped to each color and expressed with various scale. (A) 1 second per square, (B) 10 seconds per square, (C) 30 seconds per square, (D) recording the direction of scope’s movement and cecum time-location information on SRCV.

The movement of the scope, shown in Fig. 4 as a graph, was changed to a color code to increase visibility. Insertion, withdrawal, and stop were mapped to each color and expressed in 1 s, as shown in Fig. 6A. Again, the scale was changed to a 10 s scale, 30 s scale to increase visibility (Figs. 6B, 6C). One 10 s square represents 10 s. One 30 s square represents 30 s.

At the 1 s scale, the result was too microscopic and not suitable for intuitive observation. However, since the outlier belonging to a small number was removed from the 10 s scale, the movement of the scope and the position of the cecum could be intuitively grasped. In addition, as the scale increased, the distinction between the insertion phase and the withdrawal phase became clearer.

## Discussion

In this study, we developed and verified a system that can detect the direction of scope’s movement from colonoscopy video using Horn–Schunk algorithm by applying CNN deep learning methods. The motion change of the video is extracted through the Horn–Schunk algorithm for calculating optical flow. Through this algorithm, we can compute the pixel’s motion change between consecutive frames, and the direction of scope’s movement was trained and determined through the CNN. The extracted information about the direction of scope’s movement was visualized and added to the summary report of colonoscopy video (SRCV). In addition, the time-location of the cecum was calculated based on the results of direction of the scope’s movement and included in the SRCV. This proposed system can assist physician’s observation of the colonoscopy video and provide helpful information.

The videos used in this research were favorable for image acquisition and image processing because skilled physician performed the colonoscopy. This may have resulted in lowering the false-negative results when the system was applied to the video. In a previous study, we extracted 1 frame of images in 0.3 s from a colonoscopy video. The higher the extraction frequency, the more frames can be extracted, so more images can be used in the system. However, in applying the Horn-Schunck algorithm in this study, we extracted one frame at 0.5 s. Note that the Horn-Schunck algorithm can only calculate motion vectors for small changes (Meinhardt-Llopis, Pérez & Kondermann, 2013; Horn & Schunck, 1981). Therefore, if the interval between frames is too large, Horn-Schunck algorithm is not applicable. However, to calculate the motion vector based on the difference between the previous frame and the current frame, there should be a proper change between the two frames. In our colonoscopy video, a 0.5 s period yielded optimal results. As the standard setting, observing the colonoscopy video at a speed of 0.7 times to avoid misreading directions, participation of five fellow doctors, and selecting values more than three made the accuracy of the gold standard more reliable (Cho et al., 2016; Fallah & Niakan Kalhori, 2017; Cho et al., 2017)

In previous studies, we have classified informative frames and non-informative frames from the colonoscopy video with high accuracy (Cho et al., 2018). The SVM was used as it is suitable for image classification (Park, Jang & Yoo, 2016). The criteria for classification of informative frame and non-informative frame were as follows: presence of noise such as color separation phenomenon, blur caused by motion, and non-observable screen such as excessive darkness, brightness, and enlarged screen (Ballesteros et al., 2016). The mean, variance, skewness, correlation, contrast, energy of Laplacian, and energy of gradient values were acquired from the decomposed frames, and these values were used as features of the SVM model to classify the informative and non-informative frames (Sonka, Hlavac & Boyle, 2014; Hua, Fu-Long & Li-Cheng, 2006). SVM modeling was performed with the 5-fold cross-validation method.

We did not consider real-time use in this study. This is because the movement of the scope is meaningful when analyzed with the whole video. When the insertion and withdrawal movements of the scope throughout the video are analyzed, a clear distinction between the insertion phase and the withdrawal phase can be obtained. As a result, the position of the cecum can be accurately calculated.

In the methods section, we used the certain range value “t” to find the real cecum point accurately. When analyzing 112 patient images, we found numerous light insertion-withdrawal repeat movements. With the exception of polypectomy, the scopes frequently repeat insertion-withdrawal movements to find the polyps (Anderson et al., 2016). In addition, withdrawal movement was often accompanied by smooth insertion (Simmons et al., 2006). Most repetitive movements did not exceed 10 s. In other words, if “t” is less than 10 s, light repetitive insertion-withdrawal movements may have a minimum area value, so “t” is set to a sufficiently large value. Considering this, we analyzed the results when t values were 10, 20, and 30 s.

In this study, we used the CNN deep learning method to classify the movement of the scope. The images used for deep learning input were the Horn-Schunck algorithm-applied images, and the motion vector was expressed in color. In the images, the layer did not have to be deep because the directions were classified according to the color distribution only. The CNN architecture used in this study was developed with reference to LeNET and VGG, and the layer structure was experimentally optimized. The trained network was validated using the 5-fold cross-validation method, and overfitting did not occur. As a limitation of this study, we did not test data from outside the hospital. In a future study, we plan to conduct research and testing with independent data from other hospitals.

During colonoscopy, the CIT was recorded by a physician. However, as the technology is under development today, the trend is to automatically store the data generated during the medical procedure, and the information generated during colonoscopy also needs to be automatically analyzed and stored (Münzer, Schoeffmann & Böszörmenyi, 2018; Yuan, Li & Meng, 2016; Greenhalgh et al., 2010; Taira, Soderland & Jakobovits, 2001; Terada, 2015). In our previous study, we have already developed a system that automatically analyzes and records colonoscopy videos, and through the system, the video was classified by types and visualized as summary report to communicate meaning effectively (Cho et al., 2018). In addition to the previous research, it will be possible to provide more useful information to physicians via recording the direction of scope’s movement and cecum time-location information (Fig. 6D).

In this study, only the insertion, withdrawal, and stop phases were analyzed and determined. Through FGI, gastroenterologists have expressed the need to identify and visualize the anatomical structure of the large intestine. In a future study, if left and right movements can be recognized, the direction of the colonoscope can be recorded in more detail. Based on this, it is expected that the anatomical structure of the large intestine can be deduced and the anatomical position with time can be indicated.

## Conclusions

Information obtained in this study can be utilized as metadata for proficiency assessment. Since insertion and withdrawal are technically different movements, data of scope’s movement and phase can be quantified and utilized to express pattern unique to the colonoscopist and to assess proficiency (Taber & Romagnuolo, 2010; Snyder et al., 2010; Benson et al., 2010). When viewed with reference to the SRCV, we thought that the colonoscopist might have limited proficiency if there were repetitive insertion-withdrawal movements or no movements at all, which was not related to polyps, bleeding, or polypectomy.

With the proposed system, we believe that if the current handwritten medical records can be automatically summarized together with more detailed, graphical information, it will be useful for physicians and patients and will improve medical services (Ahn, Choi & Kim, 2016; Denny et al., 2010). The results of this study may contribute to improving the medical record after colonoscopy has finished. We hope that the findings of this study can contribute to the informatics field of medical records so that medical charts can be transmitted graphically and effectively in the field of colonoscopy.

##  Supplemental Information

10.7717/peerj.7256/supp-1 Dataset S1 Raw data file_Colonoscopy image Original frame image (raw data) was extracted from colonoscopy video.
In this study, colonoscopy was performed using a high-resolution colonoscopy device (CV260SL, Olympus, Tokyo, Japan). Colonoscopy videos were acquired using a video capture card (SkyCaputre U6T, Skydigital, Yongsan, Korea), after signal branching from the CV260SL. The video was converted to an MP4 format to avoid alteration of the resolution, and the resolution was 1920*1080, 30fps. The colonoscopy video was decomposed into frames. A frame was extracted as a PNG file per 0.5 s using Virtualdub software.

10.7717/peerj.7256/supp-2 Dataset S2 Raw data file_Colonoscopy image Original frame image (raw data) was extracted from colonoscopy video.
In this study, colonoscopy was performed using a high-resolution colonoscopy device (CV260SL, Olympus, Tokyo, Japan). Colonoscopy videos were acquired using a video capture card (SkyCaputre U6T, Skydigital, Yongsan, Korea), after signal branching from the CV260SL. The video was converted to an MP4 format to avoid alteration of the resolution, and the resolution was 1920*1080, 30fps. The colonoscopy video was decomposed into frames. A frame was extracted as a PNG file per 0.5 s using Virtualdub software.

10.7717/peerj.7256/supp-3 Supplemental Information 1 Clinical records of patient colonoscopy results

## Additional Information and Declarations

### Data Availability

The following information was supplied regarding data availability: The raw data is available at Figshare: Cho, Minwoo (2019): Colonoscopy images. figshare. Dataset. https://doi.org/10.6084/m9.figshare.7937336.v1.
