# Supplementary figures and images for: Identification of cecum time-location in a colonoscopy video by deep learning analysis of colonoscope movement (part 3 of 4)
Source: PeerJ. 2019 Jul 29;7:e7256. doi: 10.7717/peerj.7256 (PMC6673422; doi:10.7717/peerj.7256)

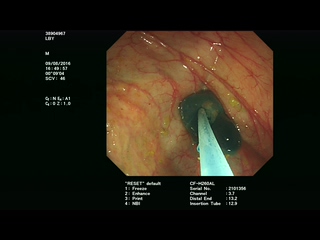

Supplement: Dataset S2 — Original frame image (raw data) was extracted from colonoscopy video. In this study, colonoscopy was performed using a high-resolution colonoscopy device (CV260SL, Olympus, Tokyo, Japan). Colonoscopy videos were acquired using a video capture card (SkyCaputre U6T, Skydigital, Yongsan, Korea), after signal branching from the CV260SL. The video was converted to an MP4 format to avoid alteration of the resolution, and the resolution was 1920*1080, 30fps. The colonoscopy video was decomposed into frames. A frame was extracted as a PNG file per 0.5 s using Virtualdub software. [file peerj-07-7256-s002.zip › f_3639.jpg]

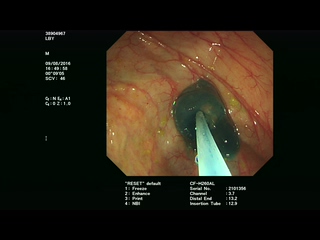

Supplement: Dataset S2 — Original frame image (raw data) was extracted from colonoscopy video. In this study, colonoscopy was performed using a high-resolution colonoscopy device (CV260SL, Olympus, Tokyo, Japan). Colonoscopy videos were acquired using a video capture card (SkyCaputre U6T, Skydigital, Yongsan, Korea), after signal branching from the CV260SL. The video was converted to an MP4 format to avoid alteration of the resolution, and the resolution was 1920*1080, 30fps. The colonoscopy video was decomposed into frames. A frame was extracted as a PNG file per 0.5 s using Virtualdub software. [file peerj-07-7256-s002.zip › f_3640.jpg]

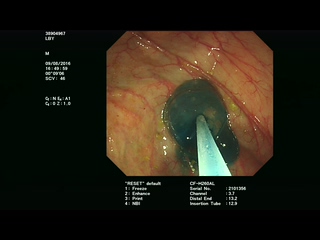

Supplement: Dataset S2 — Original frame image (raw data) was extracted from colonoscopy video. In this study, colonoscopy was performed using a high-resolution colonoscopy device (CV260SL, Olympus, Tokyo, Japan). Colonoscopy videos were acquired using a video capture card (SkyCaputre U6T, Skydigital, Yongsan, Korea), after signal branching from the CV260SL. The video was converted to an MP4 format to avoid alteration of the resolution, and the resolution was 1920*1080, 30fps. The colonoscopy video was decomposed into frames. A frame was extracted as a PNG file per 0.5 s using Virtualdub software. [file peerj-07-7256-s002.zip › f_3641.jpg]

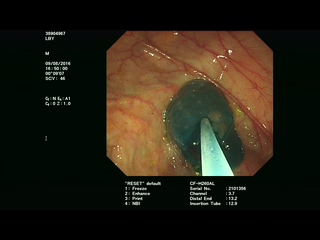

Supplement: Dataset S2 — Original frame image (raw data) was extracted from colonoscopy video. In this study, colonoscopy was performed using a high-resolution colonoscopy device (CV260SL, Olympus, Tokyo, Japan). Colonoscopy videos were acquired using a video capture card (SkyCaputre U6T, Skydigital, Yongsan, Korea), after signal branching from the CV260SL. The video was converted to an MP4 format to avoid alteration of the resolution, and the resolution was 1920*1080, 30fps. The colonoscopy video was decomposed into frames. A frame was extracted as a PNG file per 0.5 s using Virtualdub software. [file peerj-07-7256-s002.zip › f_3642.jpg]

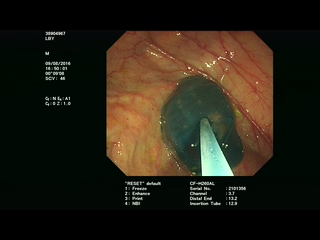

Supplement: Dataset S2 — Original frame image (raw data) was extracted from colonoscopy video. In this study, colonoscopy was performed using a high-resolution colonoscopy device (CV260SL, Olympus, Tokyo, Japan). Colonoscopy videos were acquired using a video capture card (SkyCaputre U6T, Skydigital, Yongsan, Korea), after signal branching from the CV260SL. The video was converted to an MP4 format to avoid alteration of the resolution, and the resolution was 1920*1080, 30fps. The colonoscopy video was decomposed into frames. A frame was extracted as a PNG file per 0.5 s using Virtualdub software. [file peerj-07-7256-s002.zip › f_3643.jpg]

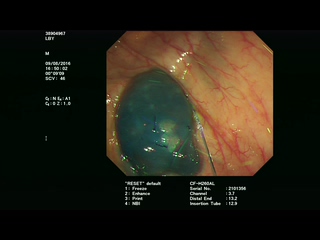

Supplement: Dataset S2 — Original frame image (raw data) was extracted from colonoscopy video. In this study, colonoscopy was performed using a high-resolution colonoscopy device (CV260SL, Olympus, Tokyo, Japan). Colonoscopy videos were acquired using a video capture card (SkyCaputre U6T, Skydigital, Yongsan, Korea), after signal branching from the CV260SL. The video was converted to an MP4 format to avoid alteration of the resolution, and the resolution was 1920*1080, 30fps. The colonoscopy video was decomposed into frames. A frame was extracted as a PNG file per 0.5 s using Virtualdub software. [file peerj-07-7256-s002.zip › f_3644.jpg]

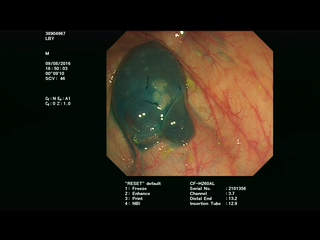

Supplement: Dataset S2 — Original frame image (raw data) was extracted from colonoscopy video. In this study, colonoscopy was performed using a high-resolution colonoscopy device (CV260SL, Olympus, Tokyo, Japan). Colonoscopy videos were acquired using a video capture card (SkyCaputre U6T, Skydigital, Yongsan, Korea), after signal branching from the CV260SL. The video was converted to an MP4 format to avoid alteration of the resolution, and the resolution was 1920*1080, 30fps. The colonoscopy video was decomposed into frames. A frame was extracted as a PNG file per 0.5 s using Virtualdub software. [file peerj-07-7256-s002.zip › f_3645.jpg]

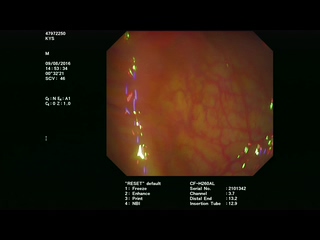

Supplement: Dataset S2 — Original frame image (raw data) was extracted from colonoscopy video. In this study, colonoscopy was performed using a high-resolution colonoscopy device (CV260SL, Olympus, Tokyo, Japan). Colonoscopy videos were acquired using a video capture card (SkyCaputre U6T, Skydigital, Yongsan, Korea), after signal branching from the CV260SL. The video was converted to an MP4 format to avoid alteration of the resolution, and the resolution was 1920*1080, 30fps. The colonoscopy video was decomposed into frames. A frame was extracted as a PNG file per 0.5 s using Virtualdub software. [file peerj-07-7256-s002.zip › f_1521.jpg]

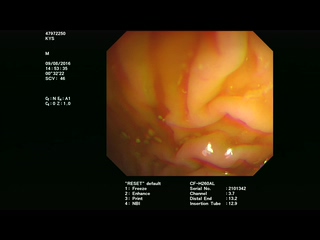

Supplement: Dataset S2 — Original frame image (raw data) was extracted from colonoscopy video. In this study, colonoscopy was performed using a high-resolution colonoscopy device (CV260SL, Olympus, Tokyo, Japan). Colonoscopy videos were acquired using a video capture card (SkyCaputre U6T, Skydigital, Yongsan, Korea), after signal branching from the CV260SL. The video was converted to an MP4 format to avoid alteration of the resolution, and the resolution was 1920*1080, 30fps. The colonoscopy video was decomposed into frames. A frame was extracted as a PNG file per 0.5 s using Virtualdub software. [file peerj-07-7256-s002.zip › f_1522.jpg]

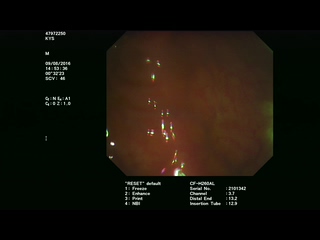

Supplement: Dataset S2 — Original frame image (raw data) was extracted from colonoscopy video. In this study, colonoscopy was performed using a high-resolution colonoscopy device (CV260SL, Olympus, Tokyo, Japan). Colonoscopy videos were acquired using a video capture card (SkyCaputre U6T, Skydigital, Yongsan, Korea), after signal branching from the CV260SL. The video was converted to an MP4 format to avoid alteration of the resolution, and the resolution was 1920*1080, 30fps. The colonoscopy video was decomposed into frames. A frame was extracted as a PNG file per 0.5 s using Virtualdub software. [file peerj-07-7256-s002.zip › f_1523.jpg]

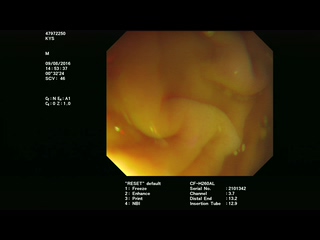

Supplement: Dataset S2 — Original frame image (raw data) was extracted from colonoscopy video. In this study, colonoscopy was performed using a high-resolution colonoscopy device (CV260SL, Olympus, Tokyo, Japan). Colonoscopy videos were acquired using a video capture card (SkyCaputre U6T, Skydigital, Yongsan, Korea), after signal branching from the CV260SL. The video was converted to an MP4 format to avoid alteration of the resolution, and the resolution was 1920*1080, 30fps. The colonoscopy video was decomposed into frames. A frame was extracted as a PNG file per 0.5 s using Virtualdub software. [file peerj-07-7256-s002.zip › f_1524.jpg]

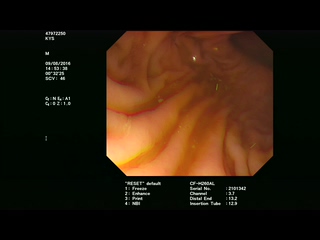

Supplement: Dataset S2 — Original frame image (raw data) was extracted from colonoscopy video. In this study, colonoscopy was performed using a high-resolution colonoscopy device (CV260SL, Olympus, Tokyo, Japan). Colonoscopy videos were acquired using a video capture card (SkyCaputre U6T, Skydigital, Yongsan, Korea), after signal branching from the CV260SL. The video was converted to an MP4 format to avoid alteration of the resolution, and the resolution was 1920*1080, 30fps. The colonoscopy video was decomposed into frames. A frame was extracted as a PNG file per 0.5 s using Virtualdub software. [file peerj-07-7256-s002.zip › f_1525.jpg]

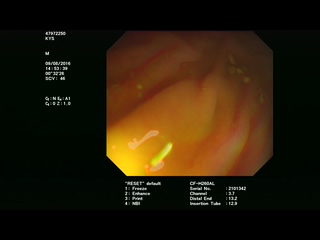

Supplement: Dataset S2 — Original frame image (raw data) was extracted from colonoscopy video. In this study, colonoscopy was performed using a high-resolution colonoscopy device (CV260SL, Olympus, Tokyo, Japan). Colonoscopy videos were acquired using a video capture card (SkyCaputre U6T, Skydigital, Yongsan, Korea), after signal branching from the CV260SL. The video was converted to an MP4 format to avoid alteration of the resolution, and the resolution was 1920*1080, 30fps. The colonoscopy video was decomposed into frames. A frame was extracted as a PNG file per 0.5 s using Virtualdub software. [file peerj-07-7256-s002.zip › f_1526.jpg]

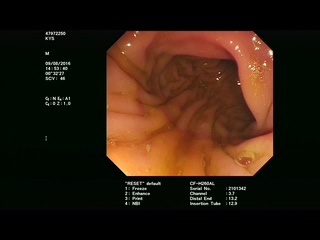

Supplement: Dataset S2 — Original frame image (raw data) was extracted from colonoscopy video. In this study, colonoscopy was performed using a high-resolution colonoscopy device (CV260SL, Olympus, Tokyo, Japan). Colonoscopy videos were acquired using a video capture card (SkyCaputre U6T, Skydigital, Yongsan, Korea), after signal branching from the CV260SL. The video was converted to an MP4 format to avoid alteration of the resolution, and the resolution was 1920*1080, 30fps. The colonoscopy video was decomposed into frames. A frame was extracted as a PNG file per 0.5 s using Virtualdub software. [file peerj-07-7256-s002.zip › f_1527.jpg]

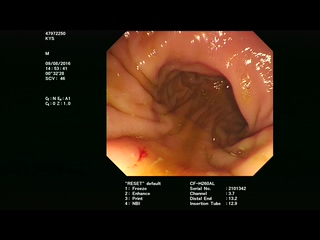

Supplement: Dataset S2 — Original frame image (raw data) was extracted from colonoscopy video. In this study, colonoscopy was performed using a high-resolution colonoscopy device (CV260SL, Olympus, Tokyo, Japan). Colonoscopy videos were acquired using a video capture card (SkyCaputre U6T, Skydigital, Yongsan, Korea), after signal branching from the CV260SL. The video was converted to an MP4 format to avoid alteration of the resolution, and the resolution was 1920*1080, 30fps. The colonoscopy video was decomposed into frames. A frame was extracted as a PNG file per 0.5 s using Virtualdub software. [file peerj-07-7256-s002.zip › f_1528.jpg]

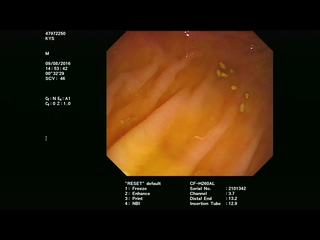

Supplement: Dataset S2 — Original frame image (raw data) was extracted from colonoscopy video. In this study, colonoscopy was performed using a high-resolution colonoscopy device (CV260SL, Olympus, Tokyo, Japan). Colonoscopy videos were acquired using a video capture card (SkyCaputre U6T, Skydigital, Yongsan, Korea), after signal branching from the CV260SL. The video was converted to an MP4 format to avoid alteration of the resolution, and the resolution was 1920*1080, 30fps. The colonoscopy video was decomposed into frames. A frame was extracted as a PNG file per 0.5 s using Virtualdub software. [file peerj-07-7256-s002.zip › f_1529.jpg]

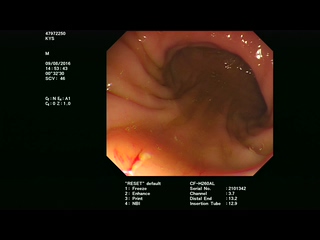

Supplement: Dataset S2 — Original frame image (raw data) was extracted from colonoscopy video. In this study, colonoscopy was performed using a high-resolution colonoscopy device (CV260SL, Olympus, Tokyo, Japan). Colonoscopy videos were acquired using a video capture card (SkyCaputre U6T, Skydigital, Yongsan, Korea), after signal branching from the CV260SL. The video was converted to an MP4 format to avoid alteration of the resolution, and the resolution was 1920*1080, 30fps. The colonoscopy video was decomposed into frames. A frame was extracted as a PNG file per 0.5 s using Virtualdub software. [file peerj-07-7256-s002.zip › f_1530.jpg]

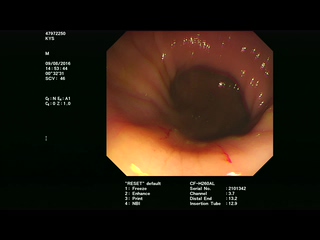

Supplement: Dataset S2 — Original frame image (raw data) was extracted from colonoscopy video. In this study, colonoscopy was performed using a high-resolution colonoscopy device (CV260SL, Olympus, Tokyo, Japan). Colonoscopy videos were acquired using a video capture card (SkyCaputre U6T, Skydigital, Yongsan, Korea), after signal branching from the CV260SL. The video was converted to an MP4 format to avoid alteration of the resolution, and the resolution was 1920*1080, 30fps. The colonoscopy video was decomposed into frames. A frame was extracted as a PNG file per 0.5 s using Virtualdub software. [file peerj-07-7256-s002.zip › f_1531.jpg]

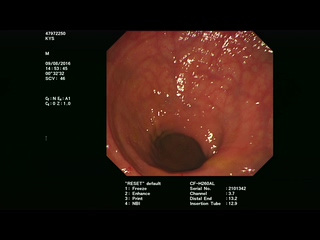

Supplement: Dataset S2 — Original frame image (raw data) was extracted from colonoscopy video. In this study, colonoscopy was performed using a high-resolution colonoscopy device (CV260SL, Olympus, Tokyo, Japan). Colonoscopy videos were acquired using a video capture card (SkyCaputre U6T, Skydigital, Yongsan, Korea), after signal branching from the CV260SL. The video was converted to an MP4 format to avoid alteration of the resolution, and the resolution was 1920*1080, 30fps. The colonoscopy video was decomposed into frames. A frame was extracted as a PNG file per 0.5 s using Virtualdub software. [file peerj-07-7256-s002.zip › f_1532.jpg]

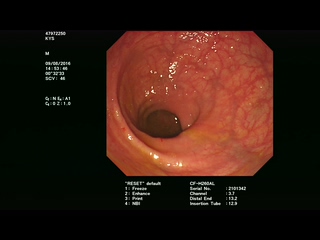

Supplement: Dataset S2 — Original frame image (raw data) was extracted from colonoscopy video. In this study, colonoscopy was performed using a high-resolution colonoscopy device (CV260SL, Olympus, Tokyo, Japan). Colonoscopy videos were acquired using a video capture card (SkyCaputre U6T, Skydigital, Yongsan, Korea), after signal branching from the CV260SL. The video was converted to an MP4 format to avoid alteration of the resolution, and the resolution was 1920*1080, 30fps. The colonoscopy video was decomposed into frames. A frame was extracted as a PNG file per 0.5 s using Virtualdub software. [file peerj-07-7256-s002.zip › f_1533.jpg]

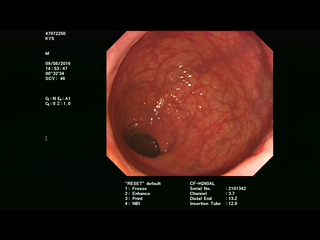

Supplement: Dataset S2 — Original frame image (raw data) was extracted from colonoscopy video. In this study, colonoscopy was performed using a high-resolution colonoscopy device (CV260SL, Olympus, Tokyo, Japan). Colonoscopy videos were acquired using a video capture card (SkyCaputre U6T, Skydigital, Yongsan, Korea), after signal branching from the CV260SL. The video was converted to an MP4 format to avoid alteration of the resolution, and the resolution was 1920*1080, 30fps. The colonoscopy video was decomposed into frames. A frame was extracted as a PNG file per 0.5 s using Virtualdub software. [file peerj-07-7256-s002.zip › f_1534.jpg]

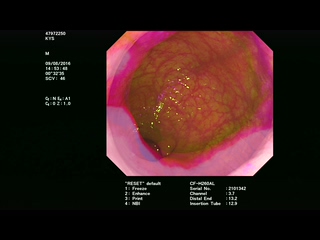

Supplement: Dataset S2 — Original frame image (raw data) was extracted from colonoscopy video. In this study, colonoscopy was performed using a high-resolution colonoscopy device (CV260SL, Olympus, Tokyo, Japan). Colonoscopy videos were acquired using a video capture card (SkyCaputre U6T, Skydigital, Yongsan, Korea), after signal branching from the CV260SL. The video was converted to an MP4 format to avoid alteration of the resolution, and the resolution was 1920*1080, 30fps. The colonoscopy video was decomposed into frames. A frame was extracted as a PNG file per 0.5 s using Virtualdub software. [file peerj-07-7256-s002.zip › f_1535.jpg]

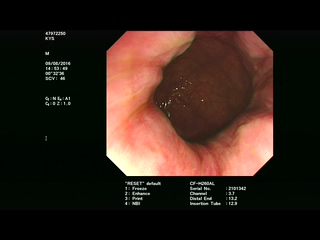

Supplement: Dataset S2 — Original frame image (raw data) was extracted from colonoscopy video. In this study, colonoscopy was performed using a high-resolution colonoscopy device (CV260SL, Olympus, Tokyo, Japan). Colonoscopy videos were acquired using a video capture card (SkyCaputre U6T, Skydigital, Yongsan, Korea), after signal branching from the CV260SL. The video was converted to an MP4 format to avoid alteration of the resolution, and the resolution was 1920*1080, 30fps. The colonoscopy video was decomposed into frames. A frame was extracted as a PNG file per 0.5 s using Virtualdub software. [file peerj-07-7256-s002.zip › f_1536.jpg]

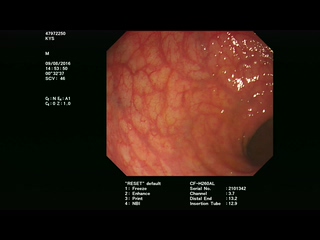

Supplement: Dataset S2 — Original frame image (raw data) was extracted from colonoscopy video. In this study, colonoscopy was performed using a high-resolution colonoscopy device (CV260SL, Olympus, Tokyo, Japan). Colonoscopy videos were acquired using a video capture card (SkyCaputre U6T, Skydigital, Yongsan, Korea), after signal branching from the CV260SL. The video was converted to an MP4 format to avoid alteration of the resolution, and the resolution was 1920*1080, 30fps. The colonoscopy video was decomposed into frames. A frame was extracted as a PNG file per 0.5 s using Virtualdub software. [file peerj-07-7256-s002.zip › f_1537.jpg]

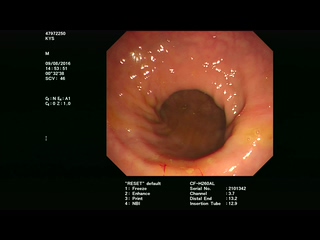

Supplement: Dataset S2 — Original frame image (raw data) was extracted from colonoscopy video. In this study, colonoscopy was performed using a high-resolution colonoscopy device (CV260SL, Olympus, Tokyo, Japan). Colonoscopy videos were acquired using a video capture card (SkyCaputre U6T, Skydigital, Yongsan, Korea), after signal branching from the CV260SL. The video was converted to an MP4 format to avoid alteration of the resolution, and the resolution was 1920*1080, 30fps. The colonoscopy video was decomposed into frames. A frame was extracted as a PNG file per 0.5 s using Virtualdub software. [file peerj-07-7256-s002.zip › f_1538.jpg]

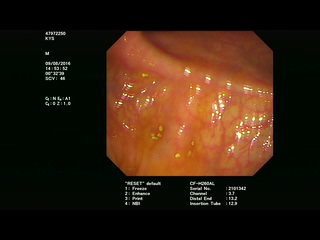

Supplement: Dataset S2 — Original frame image (raw data) was extracted from colonoscopy video. In this study, colonoscopy was performed using a high-resolution colonoscopy device (CV260SL, Olympus, Tokyo, Japan). Colonoscopy videos were acquired using a video capture card (SkyCaputre U6T, Skydigital, Yongsan, Korea), after signal branching from the CV260SL. The video was converted to an MP4 format to avoid alteration of the resolution, and the resolution was 1920*1080, 30fps. The colonoscopy video was decomposed into frames. A frame was extracted as a PNG file per 0.5 s using Virtualdub software. [file peerj-07-7256-s002.zip › f_1539.jpg]

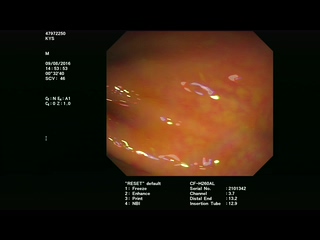

Supplement: Dataset S2 — Original frame image (raw data) was extracted from colonoscopy video. In this study, colonoscopy was performed using a high-resolution colonoscopy device (CV260SL, Olympus, Tokyo, Japan). Colonoscopy videos were acquired using a video capture card (SkyCaputre U6T, Skydigital, Yongsan, Korea), after signal branching from the CV260SL. The video was converted to an MP4 format to avoid alteration of the resolution, and the resolution was 1920*1080, 30fps. The colonoscopy video was decomposed into frames. A frame was extracted as a PNG file per 0.5 s using Virtualdub software. [file peerj-07-7256-s002.zip › f_1540.jpg]

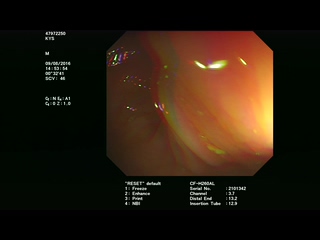

Supplement: Dataset S2 — Original frame image (raw data) was extracted from colonoscopy video. In this study, colonoscopy was performed using a high-resolution colonoscopy device (CV260SL, Olympus, Tokyo, Japan). Colonoscopy videos were acquired using a video capture card (SkyCaputre U6T, Skydigital, Yongsan, Korea), after signal branching from the CV260SL. The video was converted to an MP4 format to avoid alteration of the resolution, and the resolution was 1920*1080, 30fps. The colonoscopy video was decomposed into frames. A frame was extracted as a PNG file per 0.5 s using Virtualdub software. [file peerj-07-7256-s002.zip › f_1541.jpg]

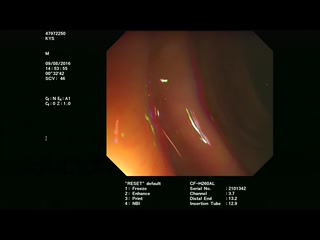

Supplement: Dataset S2 — Original frame image (raw data) was extracted from colonoscopy video. In this study, colonoscopy was performed using a high-resolution colonoscopy device (CV260SL, Olympus, Tokyo, Japan). Colonoscopy videos were acquired using a video capture card (SkyCaputre U6T, Skydigital, Yongsan, Korea), after signal branching from the CV260SL. The video was converted to an MP4 format to avoid alteration of the resolution, and the resolution was 1920*1080, 30fps. The colonoscopy video was decomposed into frames. A frame was extracted as a PNG file per 0.5 s using Virtualdub software. [file peerj-07-7256-s002.zip › f_1542.jpg]

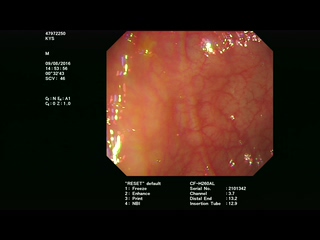

Supplement: Dataset S2 — Original frame image (raw data) was extracted from colonoscopy video. In this study, colonoscopy was performed using a high-resolution colonoscopy device (CV260SL, Olympus, Tokyo, Japan). Colonoscopy videos were acquired using a video capture card (SkyCaputre U6T, Skydigital, Yongsan, Korea), after signal branching from the CV260SL. The video was converted to an MP4 format to avoid alteration of the resolution, and the resolution was 1920*1080, 30fps. The colonoscopy video was decomposed into frames. A frame was extracted as a PNG file per 0.5 s using Virtualdub software. [file peerj-07-7256-s002.zip › f_1543.jpg]

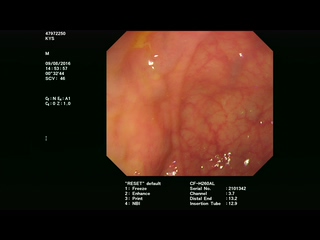

Supplement: Dataset S2 — Original frame image (raw data) was extracted from colonoscopy video. In this study, colonoscopy was performed using a high-resolution colonoscopy device (CV260SL, Olympus, Tokyo, Japan). Colonoscopy videos were acquired using a video capture card (SkyCaputre U6T, Skydigital, Yongsan, Korea), after signal branching from the CV260SL. The video was converted to an MP4 format to avoid alteration of the resolution, and the resolution was 1920*1080, 30fps. The colonoscopy video was decomposed into frames. A frame was extracted as a PNG file per 0.5 s using Virtualdub software. [file peerj-07-7256-s002.zip › f_1544.jpg]

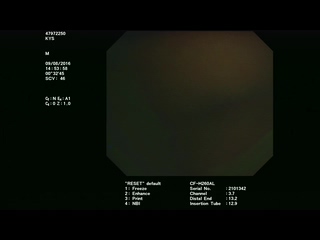

Supplement: Dataset S2 — Original frame image (raw data) was extracted from colonoscopy video. In this study, colonoscopy was performed using a high-resolution colonoscopy device (CV260SL, Olympus, Tokyo, Japan). Colonoscopy videos were acquired using a video capture card (SkyCaputre U6T, Skydigital, Yongsan, Korea), after signal branching from the CV260SL. The video was converted to an MP4 format to avoid alteration of the resolution, and the resolution was 1920*1080, 30fps. The colonoscopy video was decomposed into frames. A frame was extracted as a PNG file per 0.5 s using Virtualdub software. [file peerj-07-7256-s002.zip › f_1545.jpg]

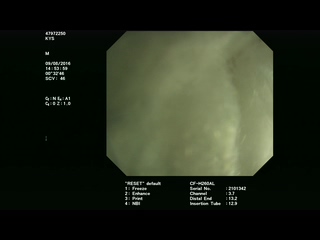

Supplement: Dataset S2 — Original frame image (raw data) was extracted from colonoscopy video. In this study, colonoscopy was performed using a high-resolution colonoscopy device (CV260SL, Olympus, Tokyo, Japan). Colonoscopy videos were acquired using a video capture card (SkyCaputre U6T, Skydigital, Yongsan, Korea), after signal branching from the CV260SL. The video was converted to an MP4 format to avoid alteration of the resolution, and the resolution was 1920*1080, 30fps. The colonoscopy video was decomposed into frames. A frame was extracted as a PNG file per 0.5 s using Virtualdub software. [file peerj-07-7256-s002.zip › f_1546.jpg]

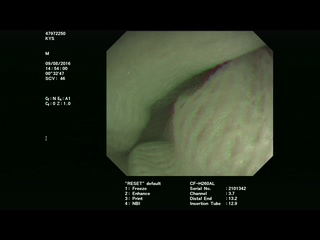

Supplement: Dataset S2 — Original frame image (raw data) was extracted from colonoscopy video. In this study, colonoscopy was performed using a high-resolution colonoscopy device (CV260SL, Olympus, Tokyo, Japan). Colonoscopy videos were acquired using a video capture card (SkyCaputre U6T, Skydigital, Yongsan, Korea), after signal branching from the CV260SL. The video was converted to an MP4 format to avoid alteration of the resolution, and the resolution was 1920*1080, 30fps. The colonoscopy video was decomposed into frames. A frame was extracted as a PNG file per 0.5 s using Virtualdub software. [file peerj-07-7256-s002.zip › f_1547.jpg]

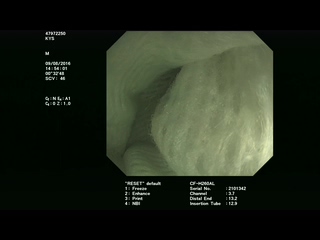

Supplement: Dataset S2 — Original frame image (raw data) was extracted from colonoscopy video. In this study, colonoscopy was performed using a high-resolution colonoscopy device (CV260SL, Olympus, Tokyo, Japan). Colonoscopy videos were acquired using a video capture card (SkyCaputre U6T, Skydigital, Yongsan, Korea), after signal branching from the CV260SL. The video was converted to an MP4 format to avoid alteration of the resolution, and the resolution was 1920*1080, 30fps. The colonoscopy video was decomposed into frames. A frame was extracted as a PNG file per 0.5 s using Virtualdub software. [file peerj-07-7256-s002.zip › f_1548.jpg]

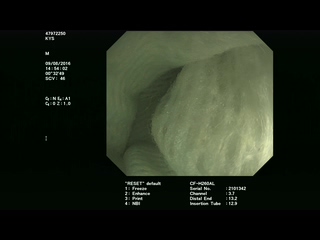

Supplement: Dataset S2 — Original frame image (raw data) was extracted from colonoscopy video. In this study, colonoscopy was performed using a high-resolution colonoscopy device (CV260SL, Olympus, Tokyo, Japan). Colonoscopy videos were acquired using a video capture card (SkyCaputre U6T, Skydigital, Yongsan, Korea), after signal branching from the CV260SL. The video was converted to an MP4 format to avoid alteration of the resolution, and the resolution was 1920*1080, 30fps. The colonoscopy video was decomposed into frames. A frame was extracted as a PNG file per 0.5 s using Virtualdub software. [file peerj-07-7256-s002.zip › f_1549.jpg]

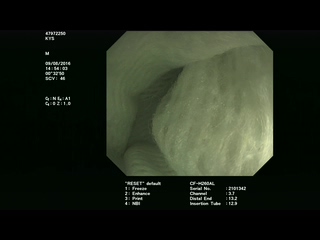

Supplement: Dataset S2 — Original frame image (raw data) was extracted from colonoscopy video. In this study, colonoscopy was performed using a high-resolution colonoscopy device (CV260SL, Olympus, Tokyo, Japan). Colonoscopy videos were acquired using a video capture card (SkyCaputre U6T, Skydigital, Yongsan, Korea), after signal branching from the CV260SL. The video was converted to an MP4 format to avoid alteration of the resolution, and the resolution was 1920*1080, 30fps. The colonoscopy video was decomposed into frames. A frame was extracted as a PNG file per 0.5 s using Virtualdub software. [file peerj-07-7256-s002.zip › f_1550.jpg]

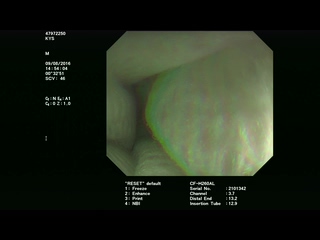

Supplement: Dataset S2 — Original frame image (raw data) was extracted from colonoscopy video. In this study, colonoscopy was performed using a high-resolution colonoscopy device (CV260SL, Olympus, Tokyo, Japan). Colonoscopy videos were acquired using a video capture card (SkyCaputre U6T, Skydigital, Yongsan, Korea), after signal branching from the CV260SL. The video was converted to an MP4 format to avoid alteration of the resolution, and the resolution was 1920*1080, 30fps. The colonoscopy video was decomposed into frames. A frame was extracted as a PNG file per 0.5 s using Virtualdub software. [file peerj-07-7256-s002.zip › f_1551.jpg]

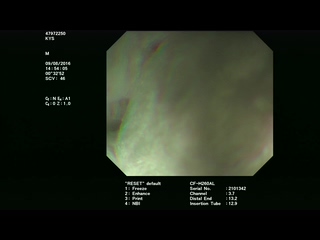

Supplement: Dataset S2 — Original frame image (raw data) was extracted from colonoscopy video. In this study, colonoscopy was performed using a high-resolution colonoscopy device (CV260SL, Olympus, Tokyo, Japan). Colonoscopy videos were acquired using a video capture card (SkyCaputre U6T, Skydigital, Yongsan, Korea), after signal branching from the CV260SL. The video was converted to an MP4 format to avoid alteration of the resolution, and the resolution was 1920*1080, 30fps. The colonoscopy video was decomposed into frames. A frame was extracted as a PNG file per 0.5 s using Virtualdub software. [file peerj-07-7256-s002.zip › f_1552.jpg]

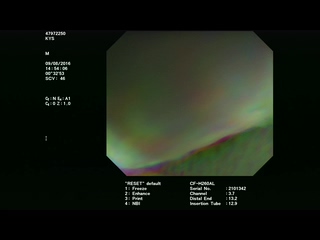

Supplement: Dataset S2 — Original frame image (raw data) was extracted from colonoscopy video. In this study, colonoscopy was performed using a high-resolution colonoscopy device (CV260SL, Olympus, Tokyo, Japan). Colonoscopy videos were acquired using a video capture card (SkyCaputre U6T, Skydigital, Yongsan, Korea), after signal branching from the CV260SL. The video was converted to an MP4 format to avoid alteration of the resolution, and the resolution was 1920*1080, 30fps. The colonoscopy video was decomposed into frames. A frame was extracted as a PNG file per 0.5 s using Virtualdub software. [file peerj-07-7256-s002.zip › f_1553.jpg]

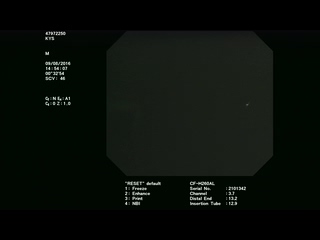

Supplement: Dataset S2 — Original frame image (raw data) was extracted from colonoscopy video. In this study, colonoscopy was performed using a high-resolution colonoscopy device (CV260SL, Olympus, Tokyo, Japan). Colonoscopy videos were acquired using a video capture card (SkyCaputre U6T, Skydigital, Yongsan, Korea), after signal branching from the CV260SL. The video was converted to an MP4 format to avoid alteration of the resolution, and the resolution was 1920*1080, 30fps. The colonoscopy video was decomposed into frames. A frame was extracted as a PNG file per 0.5 s using Virtualdub software. [file peerj-07-7256-s002.zip › f_1554.jpg]

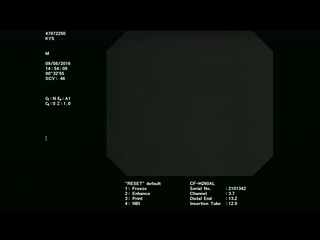

Supplement: Dataset S2 — Original frame image (raw data) was extracted from colonoscopy video. In this study, colonoscopy was performed using a high-resolution colonoscopy device (CV260SL, Olympus, Tokyo, Japan). Colonoscopy videos were acquired using a video capture card (SkyCaputre U6T, Skydigital, Yongsan, Korea), after signal branching from the CV260SL. The video was converted to an MP4 format to avoid alteration of the resolution, and the resolution was 1920*1080, 30fps. The colonoscopy video was decomposed into frames. A frame was extracted as a PNG file per 0.5 s using Virtualdub software. [file peerj-07-7256-s002.zip › f_1555.jpg]

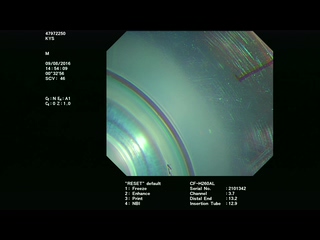

Supplement: Dataset S2 — Original frame image (raw data) was extracted from colonoscopy video. In this study, colonoscopy was performed using a high-resolution colonoscopy device (CV260SL, Olympus, Tokyo, Japan). Colonoscopy videos were acquired using a video capture card (SkyCaputre U6T, Skydigital, Yongsan, Korea), after signal branching from the CV260SL. The video was converted to an MP4 format to avoid alteration of the resolution, and the resolution was 1920*1080, 30fps. The colonoscopy video was decomposed into frames. A frame was extracted as a PNG file per 0.5 s using Virtualdub software. [file peerj-07-7256-s002.zip › f_1556.jpg]

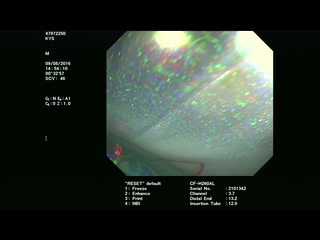

Supplement: Dataset S2 — Original frame image (raw data) was extracted from colonoscopy video. In this study, colonoscopy was performed using a high-resolution colonoscopy device (CV260SL, Olympus, Tokyo, Japan). Colonoscopy videos were acquired using a video capture card (SkyCaputre U6T, Skydigital, Yongsan, Korea), after signal branching from the CV260SL. The video was converted to an MP4 format to avoid alteration of the resolution, and the resolution was 1920*1080, 30fps. The colonoscopy video was decomposed into frames. A frame was extracted as a PNG file per 0.5 s using Virtualdub software. [file peerj-07-7256-s002.zip › f_1557.jpg]

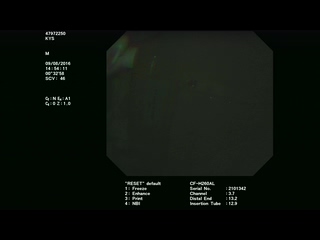

Supplement: Dataset S2 — Original frame image (raw data) was extracted from colonoscopy video. In this study, colonoscopy was performed using a high-resolution colonoscopy device (CV260SL, Olympus, Tokyo, Japan). Colonoscopy videos were acquired using a video capture card (SkyCaputre U6T, Skydigital, Yongsan, Korea), after signal branching from the CV260SL. The video was converted to an MP4 format to avoid alteration of the resolution, and the resolution was 1920*1080, 30fps. The colonoscopy video was decomposed into frames. A frame was extracted as a PNG file per 0.5 s using Virtualdub software. [file peerj-07-7256-s002.zip › f_1558.jpg]

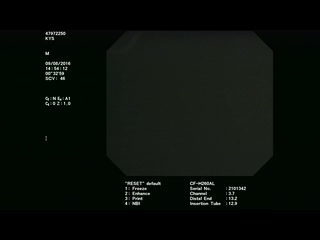

Supplement: Dataset S2 — Original frame image (raw data) was extracted from colonoscopy video. In this study, colonoscopy was performed using a high-resolution colonoscopy device (CV260SL, Olympus, Tokyo, Japan). Colonoscopy videos were acquired using a video capture card (SkyCaputre U6T, Skydigital, Yongsan, Korea), after signal branching from the CV260SL. The video was converted to an MP4 format to avoid alteration of the resolution, and the resolution was 1920*1080, 30fps. The colonoscopy video was decomposed into frames. A frame was extracted as a PNG file per 0.5 s using Virtualdub software. [file peerj-07-7256-s002.zip › f_1559.jpg]

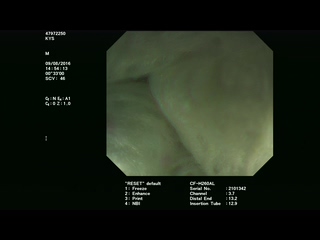

Supplement: Dataset S2 — Original frame image (raw data) was extracted from colonoscopy video. In this study, colonoscopy was performed using a high-resolution colonoscopy device (CV260SL, Olympus, Tokyo, Japan). Colonoscopy videos were acquired using a video capture card (SkyCaputre U6T, Skydigital, Yongsan, Korea), after signal branching from the CV260SL. The video was converted to an MP4 format to avoid alteration of the resolution, and the resolution was 1920*1080, 30fps. The colonoscopy video was decomposed into frames. A frame was extracted as a PNG file per 0.5 s using Virtualdub software. [file peerj-07-7256-s002.zip › f_1560.jpg]

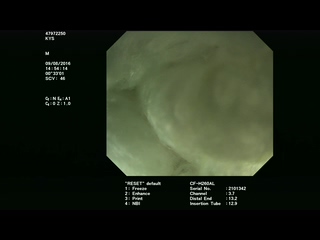

Supplement: Dataset S2 — Original frame image (raw data) was extracted from colonoscopy video. In this study, colonoscopy was performed using a high-resolution colonoscopy device (CV260SL, Olympus, Tokyo, Japan). Colonoscopy videos were acquired using a video capture card (SkyCaputre U6T, Skydigital, Yongsan, Korea), after signal branching from the CV260SL. The video was converted to an MP4 format to avoid alteration of the resolution, and the resolution was 1920*1080, 30fps. The colonoscopy video was decomposed into frames. A frame was extracted as a PNG file per 0.5 s using Virtualdub software. [file peerj-07-7256-s002.zip › f_1561.jpg]

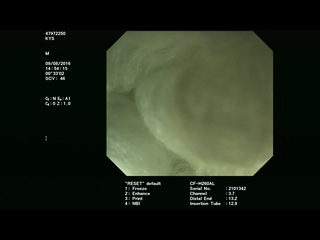

Supplement: Dataset S2 — Original frame image (raw data) was extracted from colonoscopy video. In this study, colonoscopy was performed using a high-resolution colonoscopy device (CV260SL, Olympus, Tokyo, Japan). Colonoscopy videos were acquired using a video capture card (SkyCaputre U6T, Skydigital, Yongsan, Korea), after signal branching from the CV260SL. The video was converted to an MP4 format to avoid alteration of the resolution, and the resolution was 1920*1080, 30fps. The colonoscopy video was decomposed into frames. A frame was extracted as a PNG file per 0.5 s using Virtualdub software. [file peerj-07-7256-s002.zip › f_1562.jpg]

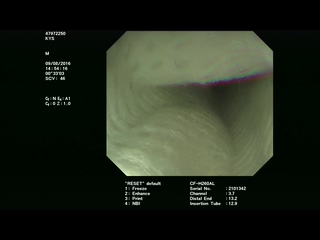

Supplement: Dataset S2 — Original frame image (raw data) was extracted from colonoscopy video. In this study, colonoscopy was performed using a high-resolution colonoscopy device (CV260SL, Olympus, Tokyo, Japan). Colonoscopy videos were acquired using a video capture card (SkyCaputre U6T, Skydigital, Yongsan, Korea), after signal branching from the CV260SL. The video was converted to an MP4 format to avoid alteration of the resolution, and the resolution was 1920*1080, 30fps. The colonoscopy video was decomposed into frames. A frame was extracted as a PNG file per 0.5 s using Virtualdub software. [file peerj-07-7256-s002.zip › f_1563.jpg]

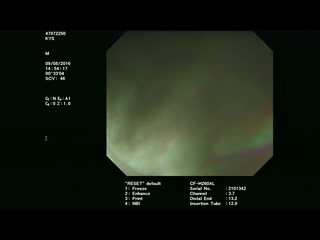

Supplement: Dataset S2 — Original frame image (raw data) was extracted from colonoscopy video. In this study, colonoscopy was performed using a high-resolution colonoscopy device (CV260SL, Olympus, Tokyo, Japan). Colonoscopy videos were acquired using a video capture card (SkyCaputre U6T, Skydigital, Yongsan, Korea), after signal branching from the CV260SL. The video was converted to an MP4 format to avoid alteration of the resolution, and the resolution was 1920*1080, 30fps. The colonoscopy video was decomposed into frames. A frame was extracted as a PNG file per 0.5 s using Virtualdub software. [file peerj-07-7256-s002.zip › f_1564.jpg]

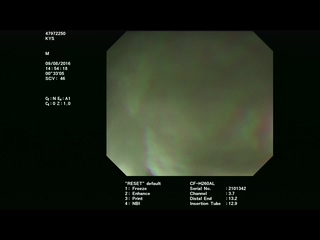

Supplement: Dataset S2 — Original frame image (raw data) was extracted from colonoscopy video. In this study, colonoscopy was performed using a high-resolution colonoscopy device (CV260SL, Olympus, Tokyo, Japan). Colonoscopy videos were acquired using a video capture card (SkyCaputre U6T, Skydigital, Yongsan, Korea), after signal branching from the CV260SL. The video was converted to an MP4 format to avoid alteration of the resolution, and the resolution was 1920*1080, 30fps. The colonoscopy video was decomposed into frames. A frame was extracted as a PNG file per 0.5 s using Virtualdub software. [file peerj-07-7256-s002.zip › f_1565.jpg]

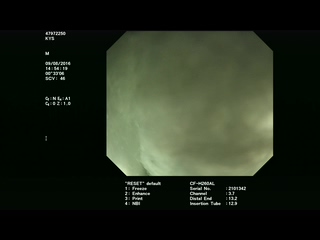

Supplement: Dataset S2 — Original frame image (raw data) was extracted from colonoscopy video. In this study, colonoscopy was performed using a high-resolution colonoscopy device (CV260SL, Olympus, Tokyo, Japan). Colonoscopy videos were acquired using a video capture card (SkyCaputre U6T, Skydigital, Yongsan, Korea), after signal branching from the CV260SL. The video was converted to an MP4 format to avoid alteration of the resolution, and the resolution was 1920*1080, 30fps. The colonoscopy video was decomposed into frames. A frame was extracted as a PNG file per 0.5 s using Virtualdub software. [file peerj-07-7256-s002.zip › f_1566.jpg]

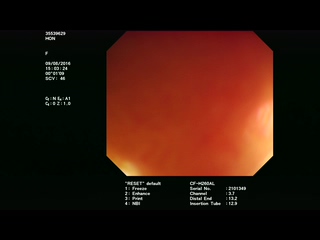

Supplement: Dataset S2 — Original frame image (raw data) was extracted from colonoscopy video. In this study, colonoscopy was performed using a high-resolution colonoscopy device (CV260SL, Olympus, Tokyo, Japan). Colonoscopy videos were acquired using a video capture card (SkyCaputre U6T, Skydigital, Yongsan, Korea), after signal branching from the CV260SL. The video was converted to an MP4 format to avoid alteration of the resolution, and the resolution was 1920*1080, 30fps. The colonoscopy video was decomposed into frames. A frame was extracted as a PNG file per 0.5 s using Virtualdub software. [file peerj-07-7256-s002.zip › f_1567.jpg]

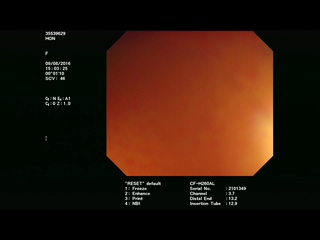

Supplement: Dataset S2 — Original frame image (raw data) was extracted from colonoscopy video. In this study, colonoscopy was performed using a high-resolution colonoscopy device (CV260SL, Olympus, Tokyo, Japan). Colonoscopy videos were acquired using a video capture card (SkyCaputre U6T, Skydigital, Yongsan, Korea), after signal branching from the CV260SL. The video was converted to an MP4 format to avoid alteration of the resolution, and the resolution was 1920*1080, 30fps. The colonoscopy video was decomposed into frames. A frame was extracted as a PNG file per 0.5 s using Virtualdub software. [file peerj-07-7256-s002.zip › f_1568.jpg]

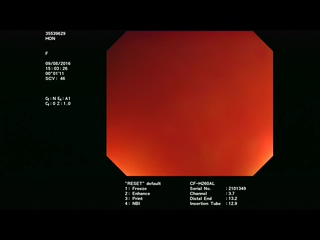

Supplement: Dataset S2 — Original frame image (raw data) was extracted from colonoscopy video. In this study, colonoscopy was performed using a high-resolution colonoscopy device (CV260SL, Olympus, Tokyo, Japan). Colonoscopy videos were acquired using a video capture card (SkyCaputre U6T, Skydigital, Yongsan, Korea), after signal branching from the CV260SL. The video was converted to an MP4 format to avoid alteration of the resolution, and the resolution was 1920*1080, 30fps. The colonoscopy video was decomposed into frames. A frame was extracted as a PNG file per 0.5 s using Virtualdub software. [file peerj-07-7256-s002.zip › f_1569.jpg]

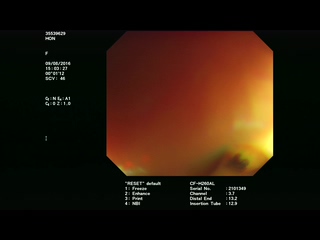

Supplement: Dataset S2 — Original frame image (raw data) was extracted from colonoscopy video. In this study, colonoscopy was performed using a high-resolution colonoscopy device (CV260SL, Olympus, Tokyo, Japan). Colonoscopy videos were acquired using a video capture card (SkyCaputre U6T, Skydigital, Yongsan, Korea), after signal branching from the CV260SL. The video was converted to an MP4 format to avoid alteration of the resolution, and the resolution was 1920*1080, 30fps. The colonoscopy video was decomposed into frames. A frame was extracted as a PNG file per 0.5 s using Virtualdub software. [file peerj-07-7256-s002.zip › f_1570.jpg]

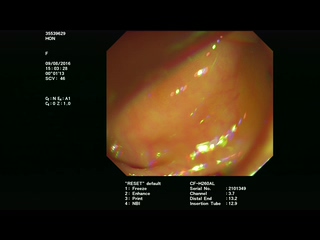

Supplement: Dataset S2 — Original frame image (raw data) was extracted from colonoscopy video. In this study, colonoscopy was performed using a high-resolution colonoscopy device (CV260SL, Olympus, Tokyo, Japan). Colonoscopy videos were acquired using a video capture card (SkyCaputre U6T, Skydigital, Yongsan, Korea), after signal branching from the CV260SL. The video was converted to an MP4 format to avoid alteration of the resolution, and the resolution was 1920*1080, 30fps. The colonoscopy video was decomposed into frames. A frame was extracted as a PNG file per 0.5 s using Virtualdub software. [file peerj-07-7256-s002.zip › f_1571.jpg]

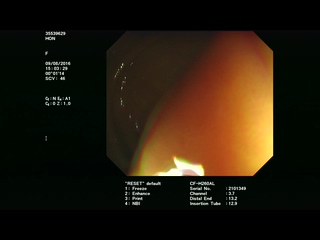

Supplement: Dataset S2 — Original frame image (raw data) was extracted from colonoscopy video. In this study, colonoscopy was performed using a high-resolution colonoscopy device (CV260SL, Olympus, Tokyo, Japan). Colonoscopy videos were acquired using a video capture card (SkyCaputre U6T, Skydigital, Yongsan, Korea), after signal branching from the CV260SL. The video was converted to an MP4 format to avoid alteration of the resolution, and the resolution was 1920*1080, 30fps. The colonoscopy video was decomposed into frames. A frame was extracted as a PNG file per 0.5 s using Virtualdub software. [file peerj-07-7256-s002.zip › f_1572.jpg]

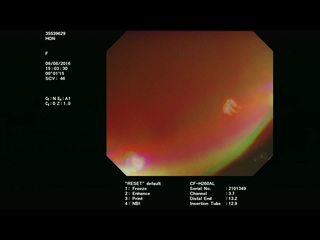

Supplement: Dataset S2 — Original frame image (raw data) was extracted from colonoscopy video. In this study, colonoscopy was performed using a high-resolution colonoscopy device (CV260SL, Olympus, Tokyo, Japan). Colonoscopy videos were acquired using a video capture card (SkyCaputre U6T, Skydigital, Yongsan, Korea), after signal branching from the CV260SL. The video was converted to an MP4 format to avoid alteration of the resolution, and the resolution was 1920*1080, 30fps. The colonoscopy video was decomposed into frames. A frame was extracted as a PNG file per 0.5 s using Virtualdub software. [file peerj-07-7256-s002.zip › f_1573.jpg]

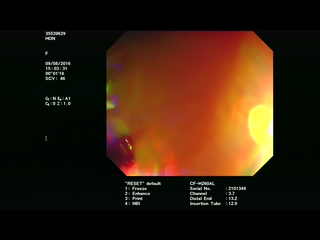

Supplement: Dataset S2 — Original frame image (raw data) was extracted from colonoscopy video. In this study, colonoscopy was performed using a high-resolution colonoscopy device (CV260SL, Olympus, Tokyo, Japan). Colonoscopy videos were acquired using a video capture card (SkyCaputre U6T, Skydigital, Yongsan, Korea), after signal branching from the CV260SL. The video was converted to an MP4 format to avoid alteration of the resolution, and the resolution was 1920*1080, 30fps. The colonoscopy video was decomposed into frames. A frame was extracted as a PNG file per 0.5 s using Virtualdub software. [file peerj-07-7256-s002.zip › f_1574.jpg]

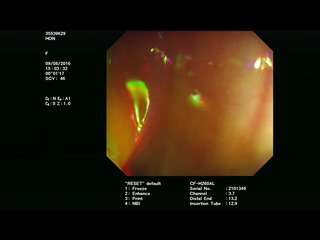

Supplement: Dataset S2 — Original frame image (raw data) was extracted from colonoscopy video. In this study, colonoscopy was performed using a high-resolution colonoscopy device (CV260SL, Olympus, Tokyo, Japan). Colonoscopy videos were acquired using a video capture card (SkyCaputre U6T, Skydigital, Yongsan, Korea), after signal branching from the CV260SL. The video was converted to an MP4 format to avoid alteration of the resolution, and the resolution was 1920*1080, 30fps. The colonoscopy video was decomposed into frames. A frame was extracted as a PNG file per 0.5 s using Virtualdub software. [file peerj-07-7256-s002.zip › f_1575.jpg]

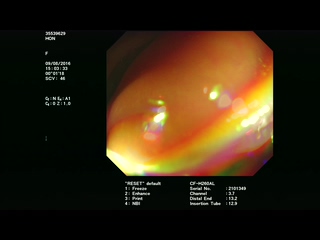

Supplement: Dataset S2 — Original frame image (raw data) was extracted from colonoscopy video. In this study, colonoscopy was performed using a high-resolution colonoscopy device (CV260SL, Olympus, Tokyo, Japan). Colonoscopy videos were acquired using a video capture card (SkyCaputre U6T, Skydigital, Yongsan, Korea), after signal branching from the CV260SL. The video was converted to an MP4 format to avoid alteration of the resolution, and the resolution was 1920*1080, 30fps. The colonoscopy video was decomposed into frames. A frame was extracted as a PNG file per 0.5 s using Virtualdub software. [file peerj-07-7256-s002.zip › f_1576.jpg]

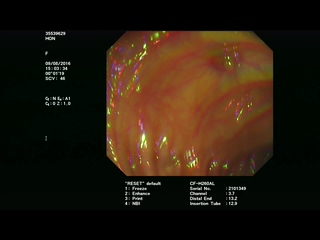

Supplement: Dataset S2 — Original frame image (raw data) was extracted from colonoscopy video. In this study, colonoscopy was performed using a high-resolution colonoscopy device (CV260SL, Olympus, Tokyo, Japan). Colonoscopy videos were acquired using a video capture card (SkyCaputre U6T, Skydigital, Yongsan, Korea), after signal branching from the CV260SL. The video was converted to an MP4 format to avoid alteration of the resolution, and the resolution was 1920*1080, 30fps. The colonoscopy video was decomposed into frames. A frame was extracted as a PNG file per 0.5 s using Virtualdub software. [file peerj-07-7256-s002.zip › f_1577.jpg]

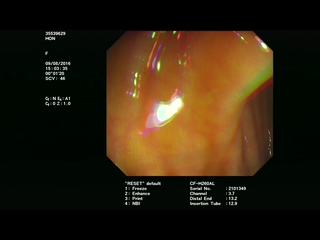

Supplement: Dataset S2 — Original frame image (raw data) was extracted from colonoscopy video. In this study, colonoscopy was performed using a high-resolution colonoscopy device (CV260SL, Olympus, Tokyo, Japan). Colonoscopy videos were acquired using a video capture card (SkyCaputre U6T, Skydigital, Yongsan, Korea), after signal branching from the CV260SL. The video was converted to an MP4 format to avoid alteration of the resolution, and the resolution was 1920*1080, 30fps. The colonoscopy video was decomposed into frames. A frame was extracted as a PNG file per 0.5 s using Virtualdub software. [file peerj-07-7256-s002.zip › f_1578.jpg]

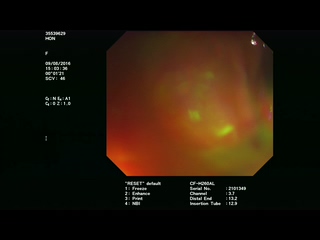

Supplement: Dataset S2 — Original frame image (raw data) was extracted from colonoscopy video. In this study, colonoscopy was performed using a high-resolution colonoscopy device (CV260SL, Olympus, Tokyo, Japan). Colonoscopy videos were acquired using a video capture card (SkyCaputre U6T, Skydigital, Yongsan, Korea), after signal branching from the CV260SL. The video was converted to an MP4 format to avoid alteration of the resolution, and the resolution was 1920*1080, 30fps. The colonoscopy video was decomposed into frames. A frame was extracted as a PNG file per 0.5 s using Virtualdub software. [file peerj-07-7256-s002.zip › f_1579.jpg]

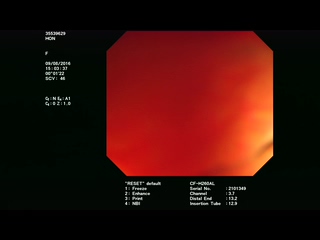

Supplement: Dataset S2 — Original frame image (raw data) was extracted from colonoscopy video. In this study, colonoscopy was performed using a high-resolution colonoscopy device (CV260SL, Olympus, Tokyo, Japan). Colonoscopy videos were acquired using a video capture card (SkyCaputre U6T, Skydigital, Yongsan, Korea), after signal branching from the CV260SL. The video was converted to an MP4 format to avoid alteration of the resolution, and the resolution was 1920*1080, 30fps. The colonoscopy video was decomposed into frames. A frame was extracted as a PNG file per 0.5 s using Virtualdub software. [file peerj-07-7256-s002.zip › f_1580.jpg]

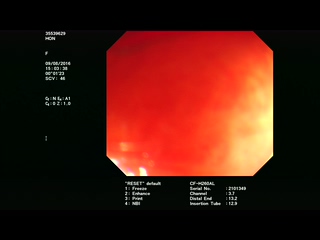

Supplement: Dataset S2 — Original frame image (raw data) was extracted from colonoscopy video. In this study, colonoscopy was performed using a high-resolution colonoscopy device (CV260SL, Olympus, Tokyo, Japan). Colonoscopy videos were acquired using a video capture card (SkyCaputre U6T, Skydigital, Yongsan, Korea), after signal branching from the CV260SL. The video was converted to an MP4 format to avoid alteration of the resolution, and the resolution was 1920*1080, 30fps. The colonoscopy video was decomposed into frames. A frame was extracted as a PNG file per 0.5 s using Virtualdub software. [file peerj-07-7256-s002.zip › f_1581.jpg]

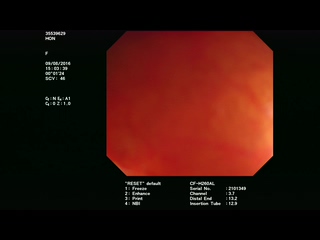

Supplement: Dataset S2 — Original frame image (raw data) was extracted from colonoscopy video. In this study, colonoscopy was performed using a high-resolution colonoscopy device (CV260SL, Olympus, Tokyo, Japan). Colonoscopy videos were acquired using a video capture card (SkyCaputre U6T, Skydigital, Yongsan, Korea), after signal branching from the CV260SL. The video was converted to an MP4 format to avoid alteration of the resolution, and the resolution was 1920*1080, 30fps. The colonoscopy video was decomposed into frames. A frame was extracted as a PNG file per 0.5 s using Virtualdub software. [file peerj-07-7256-s002.zip › f_1582.jpg]

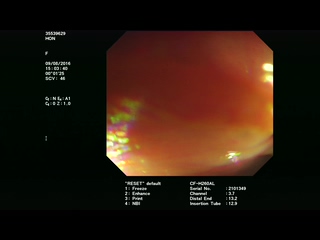

Supplement: Dataset S2 — Original frame image (raw data) was extracted from colonoscopy video. In this study, colonoscopy was performed using a high-resolution colonoscopy device (CV260SL, Olympus, Tokyo, Japan). Colonoscopy videos were acquired using a video capture card (SkyCaputre U6T, Skydigital, Yongsan, Korea), after signal branching from the CV260SL. The video was converted to an MP4 format to avoid alteration of the resolution, and the resolution was 1920*1080, 30fps. The colonoscopy video was decomposed into frames. A frame was extracted as a PNG file per 0.5 s using Virtualdub software. [file peerj-07-7256-s002.zip › f_1583.jpg]

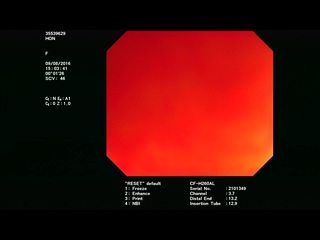

Supplement: Dataset S2 — Original frame image (raw data) was extracted from colonoscopy video. In this study, colonoscopy was performed using a high-resolution colonoscopy device (CV260SL, Olympus, Tokyo, Japan). Colonoscopy videos were acquired using a video capture card (SkyCaputre U6T, Skydigital, Yongsan, Korea), after signal branching from the CV260SL. The video was converted to an MP4 format to avoid alteration of the resolution, and the resolution was 1920*1080, 30fps. The colonoscopy video was decomposed into frames. A frame was extracted as a PNG file per 0.5 s using Virtualdub software. [file peerj-07-7256-s002.zip › f_1584.jpg]

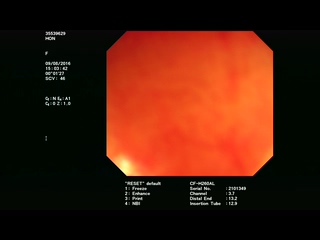

Supplement: Dataset S2 — Original frame image (raw data) was extracted from colonoscopy video. In this study, colonoscopy was performed using a high-resolution colonoscopy device (CV260SL, Olympus, Tokyo, Japan). Colonoscopy videos were acquired using a video capture card (SkyCaputre U6T, Skydigital, Yongsan, Korea), after signal branching from the CV260SL. The video was converted to an MP4 format to avoid alteration of the resolution, and the resolution was 1920*1080, 30fps. The colonoscopy video was decomposed into frames. A frame was extracted as a PNG file per 0.5 s using Virtualdub software. [file peerj-07-7256-s002.zip › f_1585.jpg]

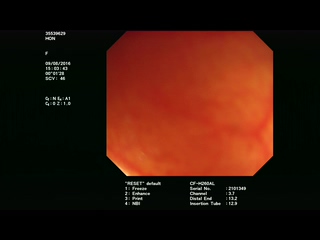

Supplement: Dataset S2 — Original frame image (raw data) was extracted from colonoscopy video. In this study, colonoscopy was performed using a high-resolution colonoscopy device (CV260SL, Olympus, Tokyo, Japan). Colonoscopy videos were acquired using a video capture card (SkyCaputre U6T, Skydigital, Yongsan, Korea), after signal branching from the CV260SL. The video was converted to an MP4 format to avoid alteration of the resolution, and the resolution was 1920*1080, 30fps. The colonoscopy video was decomposed into frames. A frame was extracted as a PNG file per 0.5 s using Virtualdub software. [file peerj-07-7256-s002.zip › f_1586.jpg]

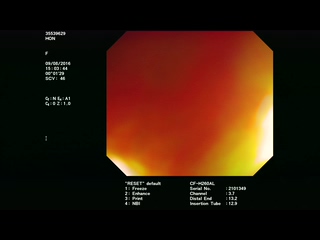

Supplement: Dataset S2 — Original frame image (raw data) was extracted from colonoscopy video. In this study, colonoscopy was performed using a high-resolution colonoscopy device (CV260SL, Olympus, Tokyo, Japan). Colonoscopy videos were acquired using a video capture card (SkyCaputre U6T, Skydigital, Yongsan, Korea), after signal branching from the CV260SL. The video was converted to an MP4 format to avoid alteration of the resolution, and the resolution was 1920*1080, 30fps. The colonoscopy video was decomposed into frames. A frame was extracted as a PNG file per 0.5 s using Virtualdub software. [file peerj-07-7256-s002.zip › f_1587.jpg]

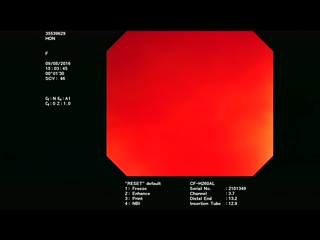

Supplement: Dataset S2 — Original frame image (raw data) was extracted from colonoscopy video. In this study, colonoscopy was performed using a high-resolution colonoscopy device (CV260SL, Olympus, Tokyo, Japan). Colonoscopy videos were acquired using a video capture card (SkyCaputre U6T, Skydigital, Yongsan, Korea), after signal branching from the CV260SL. The video was converted to an MP4 format to avoid alteration of the resolution, and the resolution was 1920*1080, 30fps. The colonoscopy video was decomposed into frames. A frame was extracted as a PNG file per 0.5 s using Virtualdub software. [file peerj-07-7256-s002.zip › f_1588.jpg]

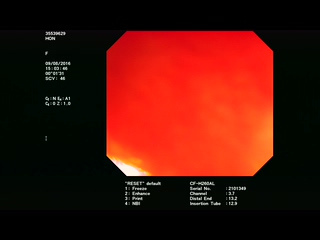

Supplement: Dataset S2 — Original frame image (raw data) was extracted from colonoscopy video. In this study, colonoscopy was performed using a high-resolution colonoscopy device (CV260SL, Olympus, Tokyo, Japan). Colonoscopy videos were acquired using a video capture card (SkyCaputre U6T, Skydigital, Yongsan, Korea), after signal branching from the CV260SL. The video was converted to an MP4 format to avoid alteration of the resolution, and the resolution was 1920*1080, 30fps. The colonoscopy video was decomposed into frames. A frame was extracted as a PNG file per 0.5 s using Virtualdub software. [file peerj-07-7256-s002.zip › f_1589.jpg]

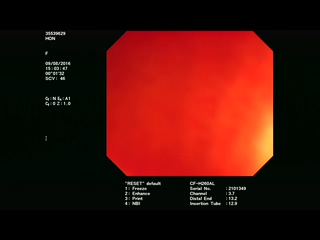

Supplement: Dataset S2 — Original frame image (raw data) was extracted from colonoscopy video. In this study, colonoscopy was performed using a high-resolution colonoscopy device (CV260SL, Olympus, Tokyo, Japan). Colonoscopy videos were acquired using a video capture card (SkyCaputre U6T, Skydigital, Yongsan, Korea), after signal branching from the CV260SL. The video was converted to an MP4 format to avoid alteration of the resolution, and the resolution was 1920*1080, 30fps. The colonoscopy video was decomposed into frames. A frame was extracted as a PNG file per 0.5 s using Virtualdub software. [file peerj-07-7256-s002.zip › f_1590.jpg]

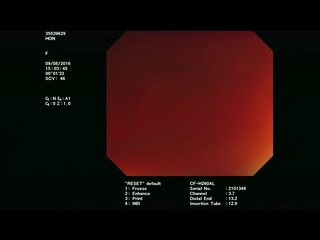

Supplement: Dataset S2 — Original frame image (raw data) was extracted from colonoscopy video. In this study, colonoscopy was performed using a high-resolution colonoscopy device (CV260SL, Olympus, Tokyo, Japan). Colonoscopy videos were acquired using a video capture card (SkyCaputre U6T, Skydigital, Yongsan, Korea), after signal branching from the CV260SL. The video was converted to an MP4 format to avoid alteration of the resolution, and the resolution was 1920*1080, 30fps. The colonoscopy video was decomposed into frames. A frame was extracted as a PNG file per 0.5 s using Virtualdub software. [file peerj-07-7256-s002.zip › f_1591.jpg]

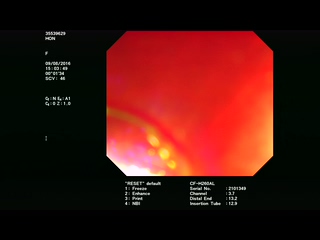

Supplement: Dataset S2 — Original frame image (raw data) was extracted from colonoscopy video. In this study, colonoscopy was performed using a high-resolution colonoscopy device (CV260SL, Olympus, Tokyo, Japan). Colonoscopy videos were acquired using a video capture card (SkyCaputre U6T, Skydigital, Yongsan, Korea), after signal branching from the CV260SL. The video was converted to an MP4 format to avoid alteration of the resolution, and the resolution was 1920*1080, 30fps. The colonoscopy video was decomposed into frames. A frame was extracted as a PNG file per 0.5 s using Virtualdub software. [file peerj-07-7256-s002.zip › f_1592.jpg]

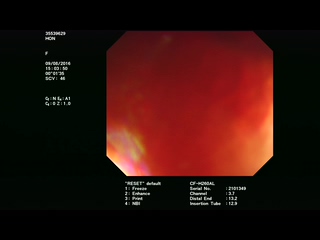

Supplement: Dataset S2 — Original frame image (raw data) was extracted from colonoscopy video. In this study, colonoscopy was performed using a high-resolution colonoscopy device (CV260SL, Olympus, Tokyo, Japan). Colonoscopy videos were acquired using a video capture card (SkyCaputre U6T, Skydigital, Yongsan, Korea), after signal branching from the CV260SL. The video was converted to an MP4 format to avoid alteration of the resolution, and the resolution was 1920*1080, 30fps. The colonoscopy video was decomposed into frames. A frame was extracted as a PNG file per 0.5 s using Virtualdub software. [file peerj-07-7256-s002.zip › f_1593.jpg]

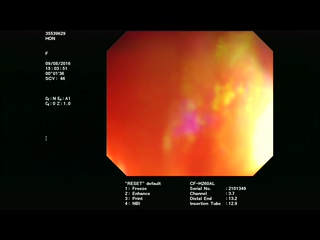

Supplement: Dataset S2 — Original frame image (raw data) was extracted from colonoscopy video. In this study, colonoscopy was performed using a high-resolution colonoscopy device (CV260SL, Olympus, Tokyo, Japan). Colonoscopy videos were acquired using a video capture card (SkyCaputre U6T, Skydigital, Yongsan, Korea), after signal branching from the CV260SL. The video was converted to an MP4 format to avoid alteration of the resolution, and the resolution was 1920*1080, 30fps. The colonoscopy video was decomposed into frames. A frame was extracted as a PNG file per 0.5 s using Virtualdub software. [file peerj-07-7256-s002.zip › f_1594.jpg]

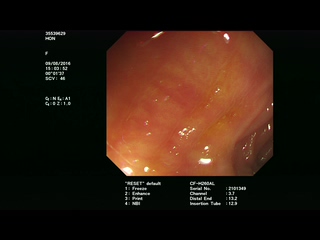

Supplement: Dataset S2 — Original frame image (raw data) was extracted from colonoscopy video. In this study, colonoscopy was performed using a high-resolution colonoscopy device (CV260SL, Olympus, Tokyo, Japan). Colonoscopy videos were acquired using a video capture card (SkyCaputre U6T, Skydigital, Yongsan, Korea), after signal branching from the CV260SL. The video was converted to an MP4 format to avoid alteration of the resolution, and the resolution was 1920*1080, 30fps. The colonoscopy video was decomposed into frames. A frame was extracted as a PNG file per 0.5 s using Virtualdub software. [file peerj-07-7256-s002.zip › f_1595.jpg]

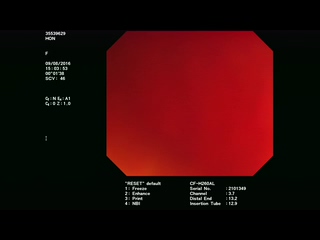

Supplement: Dataset S2 — Original frame image (raw data) was extracted from colonoscopy video. In this study, colonoscopy was performed using a high-resolution colonoscopy device (CV260SL, Olympus, Tokyo, Japan). Colonoscopy videos were acquired using a video capture card (SkyCaputre U6T, Skydigital, Yongsan, Korea), after signal branching from the CV260SL. The video was converted to an MP4 format to avoid alteration of the resolution, and the resolution was 1920*1080, 30fps. The colonoscopy video was decomposed into frames. A frame was extracted as a PNG file per 0.5 s using Virtualdub software. [file peerj-07-7256-s002.zip › f_1596.jpg]

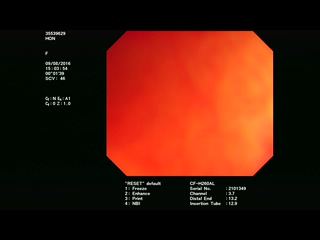

Supplement: Dataset S2 — Original frame image (raw data) was extracted from colonoscopy video. In this study, colonoscopy was performed using a high-resolution colonoscopy device (CV260SL, Olympus, Tokyo, Japan). Colonoscopy videos were acquired using a video capture card (SkyCaputre U6T, Skydigital, Yongsan, Korea), after signal branching from the CV260SL. The video was converted to an MP4 format to avoid alteration of the resolution, and the resolution was 1920*1080, 30fps. The colonoscopy video was decomposed into frames. A frame was extracted as a PNG file per 0.5 s using Virtualdub software. [file peerj-07-7256-s002.zip › f_1597.jpg]

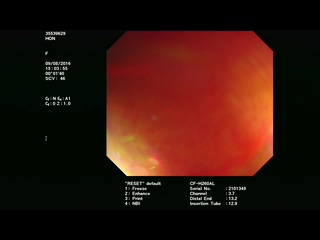

Supplement: Dataset S2 — Original frame image (raw data) was extracted from colonoscopy video. In this study, colonoscopy was performed using a high-resolution colonoscopy device (CV260SL, Olympus, Tokyo, Japan). Colonoscopy videos were acquired using a video capture card (SkyCaputre U6T, Skydigital, Yongsan, Korea), after signal branching from the CV260SL. The video was converted to an MP4 format to avoid alteration of the resolution, and the resolution was 1920*1080, 30fps. The colonoscopy video was decomposed into frames. A frame was extracted as a PNG file per 0.5 s using Virtualdub software. [file peerj-07-7256-s002.zip › f_1598.jpg]

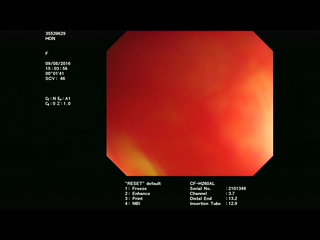

Supplement: Dataset S2 — Original frame image (raw data) was extracted from colonoscopy video. In this study, colonoscopy was performed using a high-resolution colonoscopy device (CV260SL, Olympus, Tokyo, Japan). Colonoscopy videos were acquired using a video capture card (SkyCaputre U6T, Skydigital, Yongsan, Korea), after signal branching from the CV260SL. The video was converted to an MP4 format to avoid alteration of the resolution, and the resolution was 1920*1080, 30fps. The colonoscopy video was decomposed into frames. A frame was extracted as a PNG file per 0.5 s using Virtualdub software. [file peerj-07-7256-s002.zip › f_1599.jpg]

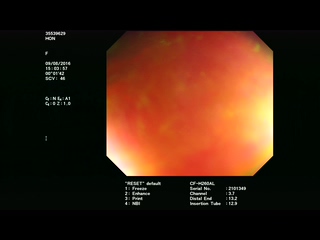

Supplement: Dataset S2 — Original frame image (raw data) was extracted from colonoscopy video. In this study, colonoscopy was performed using a high-resolution colonoscopy device (CV260SL, Olympus, Tokyo, Japan). Colonoscopy videos were acquired using a video capture card (SkyCaputre U6T, Skydigital, Yongsan, Korea), after signal branching from the CV260SL. The video was converted to an MP4 format to avoid alteration of the resolution, and the resolution was 1920*1080, 30fps. The colonoscopy video was decomposed into frames. A frame was extracted as a PNG file per 0.5 s using Virtualdub software. [file peerj-07-7256-s002.zip › f_1600.jpg]

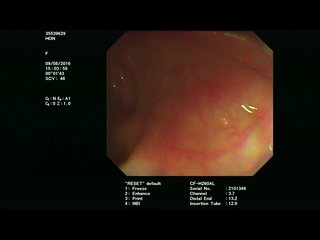

Supplement: Dataset S2 — Original frame image (raw data) was extracted from colonoscopy video. In this study, colonoscopy was performed using a high-resolution colonoscopy device (CV260SL, Olympus, Tokyo, Japan). Colonoscopy videos were acquired using a video capture card (SkyCaputre U6T, Skydigital, Yongsan, Korea), after signal branching from the CV260SL. The video was converted to an MP4 format to avoid alteration of the resolution, and the resolution was 1920*1080, 30fps. The colonoscopy video was decomposed into frames. A frame was extracted as a PNG file per 0.5 s using Virtualdub software. [file peerj-07-7256-s002.zip › f_1601.jpg]

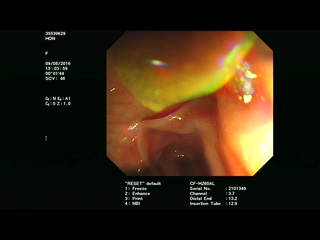

Supplement: Dataset S2 — Original frame image (raw data) was extracted from colonoscopy video. In this study, colonoscopy was performed using a high-resolution colonoscopy device (CV260SL, Olympus, Tokyo, Japan). Colonoscopy videos were acquired using a video capture card (SkyCaputre U6T, Skydigital, Yongsan, Korea), after signal branching from the CV260SL. The video was converted to an MP4 format to avoid alteration of the resolution, and the resolution was 1920*1080, 30fps. The colonoscopy video was decomposed into frames. A frame was extracted as a PNG file per 0.5 s using Virtualdub software. [file peerj-07-7256-s002.zip › f_1602.jpg]

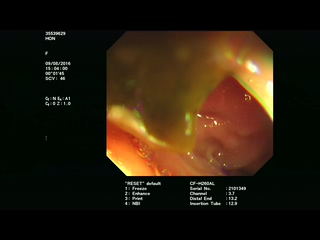

Supplement: Dataset S2 — Original frame image (raw data) was extracted from colonoscopy video. In this study, colonoscopy was performed using a high-resolution colonoscopy device (CV260SL, Olympus, Tokyo, Japan). Colonoscopy videos were acquired using a video capture card (SkyCaputre U6T, Skydigital, Yongsan, Korea), after signal branching from the CV260SL. The video was converted to an MP4 format to avoid alteration of the resolution, and the resolution was 1920*1080, 30fps. The colonoscopy video was decomposed into frames. A frame was extracted as a PNG file per 0.5 s using Virtualdub software. [file peerj-07-7256-s002.zip › f_1603.jpg]

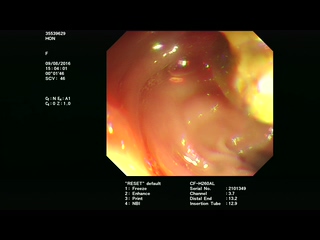

Supplement: Dataset S2 — Original frame image (raw data) was extracted from colonoscopy video. In this study, colonoscopy was performed using a high-resolution colonoscopy device (CV260SL, Olympus, Tokyo, Japan). Colonoscopy videos were acquired using a video capture card (SkyCaputre U6T, Skydigital, Yongsan, Korea), after signal branching from the CV260SL. The video was converted to an MP4 format to avoid alteration of the resolution, and the resolution was 1920*1080, 30fps. The colonoscopy video was decomposed into frames. A frame was extracted as a PNG file per 0.5 s using Virtualdub software. [file peerj-07-7256-s002.zip › f_1604.jpg]

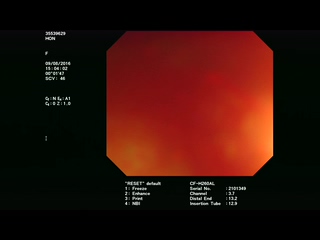

Supplement: Dataset S2 — Original frame image (raw data) was extracted from colonoscopy video. In this study, colonoscopy was performed using a high-resolution colonoscopy device (CV260SL, Olympus, Tokyo, Japan). Colonoscopy videos were acquired using a video capture card (SkyCaputre U6T, Skydigital, Yongsan, Korea), after signal branching from the CV260SL. The video was converted to an MP4 format to avoid alteration of the resolution, and the resolution was 1920*1080, 30fps. The colonoscopy video was decomposed into frames. A frame was extracted as a PNG file per 0.5 s using Virtualdub software. [file peerj-07-7256-s002.zip › f_1605.jpg]

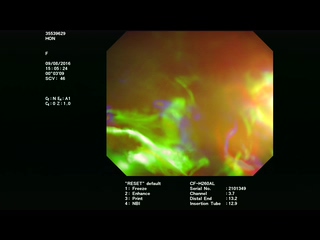

Supplement: Dataset S2 — Original frame image (raw data) was extracted from colonoscopy video. In this study, colonoscopy was performed using a high-resolution colonoscopy device (CV260SL, Olympus, Tokyo, Japan). Colonoscopy videos were acquired using a video capture card (SkyCaputre U6T, Skydigital, Yongsan, Korea), after signal branching from the CV260SL. The video was converted to an MP4 format to avoid alteration of the resolution, and the resolution was 1920*1080, 30fps. The colonoscopy video was decomposed into frames. A frame was extracted as a PNG file per 0.5 s using Virtualdub software. [file peerj-07-7256-s002.zip › f_1606.jpg]

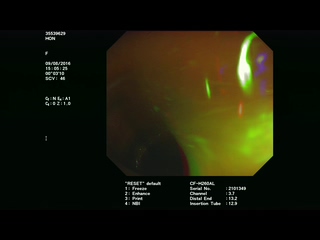

Supplement: Dataset S2 — Original frame image (raw data) was extracted from colonoscopy video. In this study, colonoscopy was performed using a high-resolution colonoscopy device (CV260SL, Olympus, Tokyo, Japan). Colonoscopy videos were acquired using a video capture card (SkyCaputre U6T, Skydigital, Yongsan, Korea), after signal branching from the CV260SL. The video was converted to an MP4 format to avoid alteration of the resolution, and the resolution was 1920*1080, 30fps. The colonoscopy video was decomposed into frames. A frame was extracted as a PNG file per 0.5 s using Virtualdub software. [file peerj-07-7256-s002.zip › f_1607.jpg]

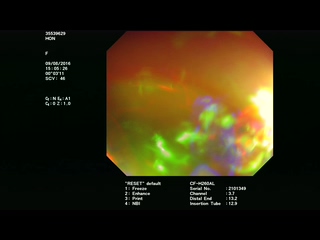

Supplement: Dataset S2 — Original frame image (raw data) was extracted from colonoscopy video. In this study, colonoscopy was performed using a high-resolution colonoscopy device (CV260SL, Olympus, Tokyo, Japan). Colonoscopy videos were acquired using a video capture card (SkyCaputre U6T, Skydigital, Yongsan, Korea), after signal branching from the CV260SL. The video was converted to an MP4 format to avoid alteration of the resolution, and the resolution was 1920*1080, 30fps. The colonoscopy video was decomposed into frames. A frame was extracted as a PNG file per 0.5 s using Virtualdub software. [file peerj-07-7256-s002.zip › f_1608.jpg]

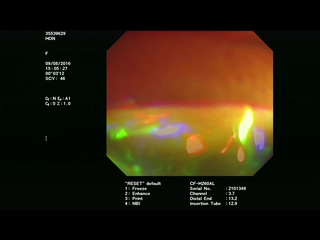

Supplement: Dataset S2 — Original frame image (raw data) was extracted from colonoscopy video. In this study, colonoscopy was performed using a high-resolution colonoscopy device (CV260SL, Olympus, Tokyo, Japan). Colonoscopy videos were acquired using a video capture card (SkyCaputre U6T, Skydigital, Yongsan, Korea), after signal branching from the CV260SL. The video was converted to an MP4 format to avoid alteration of the resolution, and the resolution was 1920*1080, 30fps. The colonoscopy video was decomposed into frames. A frame was extracted as a PNG file per 0.5 s using Virtualdub software. [file peerj-07-7256-s002.zip › f_1609.jpg]

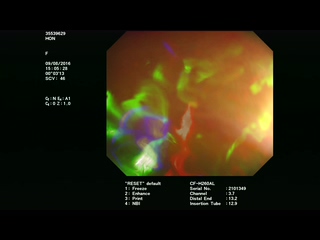

Supplement: Dataset S2 — Original frame image (raw data) was extracted from colonoscopy video. In this study, colonoscopy was performed using a high-resolution colonoscopy device (CV260SL, Olympus, Tokyo, Japan). Colonoscopy videos were acquired using a video capture card (SkyCaputre U6T, Skydigital, Yongsan, Korea), after signal branching from the CV260SL. The video was converted to an MP4 format to avoid alteration of the resolution, and the resolution was 1920*1080, 30fps. The colonoscopy video was decomposed into frames. A frame was extracted as a PNG file per 0.5 s using Virtualdub software. [file peerj-07-7256-s002.zip › f_1610.jpg]

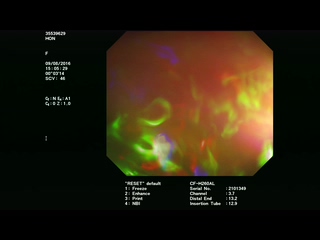

Supplement: Dataset S2 — Original frame image (raw data) was extracted from colonoscopy video. In this study, colonoscopy was performed using a high-resolution colonoscopy device (CV260SL, Olympus, Tokyo, Japan). Colonoscopy videos were acquired using a video capture card (SkyCaputre U6T, Skydigital, Yongsan, Korea), after signal branching from the CV260SL. The video was converted to an MP4 format to avoid alteration of the resolution, and the resolution was 1920*1080, 30fps. The colonoscopy video was decomposed into frames. A frame was extracted as a PNG file per 0.5 s using Virtualdub software. [file peerj-07-7256-s002.zip › f_1611.jpg]

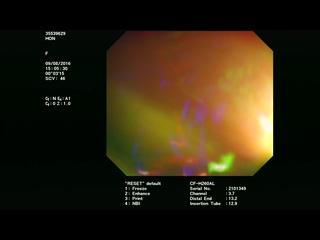

Supplement: Dataset S2 — Original frame image (raw data) was extracted from colonoscopy video. In this study, colonoscopy was performed using a high-resolution colonoscopy device (CV260SL, Olympus, Tokyo, Japan). Colonoscopy videos were acquired using a video capture card (SkyCaputre U6T, Skydigital, Yongsan, Korea), after signal branching from the CV260SL. The video was converted to an MP4 format to avoid alteration of the resolution, and the resolution was 1920*1080, 30fps. The colonoscopy video was decomposed into frames. A frame was extracted as a PNG file per 0.5 s using Virtualdub software. [file peerj-07-7256-s002.zip › f_1612.jpg]

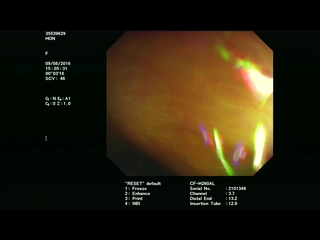

Supplement: Dataset S2 — Original frame image (raw data) was extracted from colonoscopy video. In this study, colonoscopy was performed using a high-resolution colonoscopy device (CV260SL, Olympus, Tokyo, Japan). Colonoscopy videos were acquired using a video capture card (SkyCaputre U6T, Skydigital, Yongsan, Korea), after signal branching from the CV260SL. The video was converted to an MP4 format to avoid alteration of the resolution, and the resolution was 1920*1080, 30fps. The colonoscopy video was decomposed into frames. A frame was extracted as a PNG file per 0.5 s using Virtualdub software. [file peerj-07-7256-s002.zip › f_1613.jpg]
